# A single-cell map of vascular and tissue lymphocytes identifies proliferative TCF-1^+^ human innate lymphoid cells

**DOI:** 10.3389/fimmu.2022.902881

**Published:** 2022-07-27

**Authors:** Yu Gao, Arlisa Alisjahbana, Daryl Zhong Hao Boey, Imran Mohammad, Natalie Sleiers, Joakim S. Dahlin, Tim Willinger

**Affiliations:** ^1^ Center for Infectious Medicine, Department of Medicine Huddinge, Karolinska Institutet, Karolinska University Hospital, Stockholm, Sweden; ^2^ Department of Medicine Solna, Karolinska Institutet, Karolinska University Hospital, Stockholm, Sweden

**Keywords:** innate lymphoid cells, proliferation, migration, tissue residency, ontogeny, spleen, humanized mice, single-cell RNA-sequencing

## Abstract

Innate lymphoid cells (ILCs) play important roles in tissue homeostasis and host defense, but the proliferative properties and migratory behavior of especially human ILCs remain poorly understood. Here we mapped at single-cell resolution the spatial distribution of quiescent and proliferative human ILCs within the vascular versus tissue compartment. For this purpose, we employed MISTRG humanized mice as an *in-vivo* model to study human ILCs. We uncovered subset-specific differences in the proliferative status between vascular and tissue ILCs within lymphoid and non-lymphoid organs. We also identified CD117^-^CRTH2^-^CD45RA^+^ ILCs in the spleen that were highly proliferative and expressed the transcription factor TCF-1. These proliferative ILCs were present during the neonatal period in human blood and emerged early during population of the human ILC compartment in MISTRG mice transplanted with human hematopoietic stem and progenitor cells (HSPCs). Single-cell RNA-sequencing combined with intravascular cell labeling suggested that proliferative ILCs actively migrated from the local vasculature into the spleen tissue. Collectively, our comprehensive map reveals the proliferative topography of human ILCs, linking cell migration and spatial compartmentalization with cell division.

## Introduction

ILCs belong to a new lineage of immune cells of lymphoid origin that has innate properties, such as rapid effector function (1–6). Similar to T lymphocytes, ILCs are classified into distinct subsets that express lineage-defining transcription factors and effector molecules: (i) Cytotoxic natural killer (NK) cells expressing the signature transcription factors EOMES and T-BET; (ii) ILC1s expressing T-BET and the signature cytokine interferon gamma (IFNγ); (iii) ILC2s expressing GATA3 and the cytokines interleukin-5 (IL-5) and IL-13; (iv) ILC3s expressing RORγt and the effector cytokines IL-17 and/or IL-22. Surface expression of IL-7Rα (CD127) is generally used to distinguish helper-like CD127^+^ ILC1s, ILC2s, and ILC3s from CD127^-^CD94^+^ human NK cells (7).

ILCs inhabit various organs where they are strategically positioned to help maintain organ homeostasis and to participate in host defense. ILCs are considered to be mainly tissue-resident at steady state, at least in mice (8). However, ILCs actively migrate during their ontogeny and tissue injury (9–19) and the function of ILCs is linked to their localization within tissues (20, 21). The relative frequency of human ILC subsets differs between organs (22, 23). Moreover, differences in ILC compartmentalization between mice and humans have been reported (23). However, the distribution of human ILCs between the vascular and tissue space is unknown due to the difficulty of studying human ILCs *in vivo*.

ILCs lack antigen-specific receptors but expand in response to locally produced cytokines. Recently, the concept that mature ILCs differentiate locally from systemic ILC precursors (ILCPs) that enter tissues has been established (11, 24–26). However, the mechanisms of ILC proliferation, especially in the human context, are not completely understood. For example, it is unclear where ILCs proliferate and whether ILC proliferation is spatially compartmentalized. We hypothesized that ILCs differ in their proliferative status and that it is linked to their anatomical location. To test this hypothesis, we used a humanized mouse model to investigate human ILCs *in vivo* because human ILC studies are largely restricted to *ex-vivo* and *in-vitro* experiments. This model named “MISTRG” expresses human cytokines (27–29) and supports the development of human NK cells and all types of ILCs after transplantation with human hematopoietic stem and progenitor cells (30, 31). Therefore, this model allows studying human ILCs directly in their surrounding tissue microenvironment and to investigate their ontogeny, migration, and proliferation *in vivo*.

Here, we comprehensively resolved the spatial distribution of quiescent and proliferating human ILCs in bone marrow, spleen, liver, and lung of MISTRG mice transplanted with human CD34^+^ HSPCs. Intravascular labeling to distinguish vascular from tissue ILCs revealed that human ILCs have a specific proliferative topology within lymphoid and non-lymphoid organs. Moreover, we identified a specific population of CD117^-^CRTH2^-^CD45RA^+^ ILCs with high proliferative potential that expressed the transcription factor TCF-1, emerged early during ontogeny, and acquired tissue residency in the spleen. Our findings support the notion that ILCs residing in the local organ vasculature are primed for proliferation before entering the extravascular (tissue) compartment.

## Materials and methods

### Human blood

We obtained human tissues through Karolinska University Hospital Huddinge after informed consent was given by all tissue donors following verbal and written information. Umbilical cord blood was obtained from Caesarean sections. Buffy coats were provided by the local Blood Bank. Approval for the use of human tissues was obtained from the Ethical Review Board at Karolinska Institutet (#2006/229-31/3, 2015/1368-31/4, 2015/2122-32, 2016/1415-32, 2019-03863). The study was conducted according to the Declaration of Helsinki.

### Generation of mice with a human immune system

To study human ILCs *in vivo*, we used MISTRG mice as in our recent study (31). MISTRG mice on the *Rag2* ^-/-^ *Il2rg* ^-/-^ background were previously described (27, 32) and express human genes encoding the proteins M-CSF, IL-3/GM-CSF, SIRPα, and TPO through gene knock-in. For the experiments, we used both male and female MISTRG mice that were heterozygous for *SIRPA* and homozygous for all other human genes. MISTRG mice were housed under specific pathogen-free conditions and did not receive any prophylactic antibiotics. Human CD34^+^ cells containing HSPCs were purified from umbilical cord blood (pooled from several donors) with the CD34^+^ microbead kit (Miltenyi Biotec) as previously described (31). Newborn MISTG mice that were 3-5 days old were then injected with 1 x 10^5^ human CD34^+^ cells (pooled from several donors) *via* the intrahepatic route as described (31, 33). Mice did not receive any irradiation as pre-conditioning before transplantation except for the experiments shown in Supplementary **Figure 4** and for the single-cell RNA-sequencing of spleen ILCs. In these cases, mice were irradiated once with 100 cGy before HSPC transplantation. Successful engraftment with human CD45^+^ hematopoietic cells was confirmed by flow cytometry of blood collected from MISTRG mice at ~6-8 weeks post-transplantation. In general, HSPC-engrafted MISTRG mice were used for experiments 7-12 weeks after injection with human CD34^+^ cells, except for the kinetics experiments where mice were analyzed also at 3-5 weeks post-transplantation. All mouse experiments were approved by the Linköping Animal Experimentation Ethics Committee (ethical permits #29-15 and #03127-2020). The use of MISTRG mice requires Material Transfer Agreements with Regeneron Pharmaceuticals and Yale University.

### Labeling of intravascular ILCs in MISTRG mice

Human CD45^+^ hematopoietic cells in the blood and the organ vasculature of HSPC-engrafted MISTRG mice were labeled as previously described (31). For this purpose, 2 μg of a phycoerythrin (PE)-conjugated anti-human CD45 antibody (Biolegend, clone HI30) was injected intravenously (IV) 5 minutes before organ harvest without any prior perfusion. After isolation from various organs (bone marrow, spleen, liver, lung), cells were stained *ex vivo* with an APC-Cy7-conjugated anti-human CD45 antibody and other cell surface antibodies to determine the frequency of intravascular (IV CD45-PE^+^) and extravascular (IV CD45-PE^-^) ILCs by flow cytometry. Blood was also analyzed by flow cytometry to confirm successful intravascular labelling, defined as >90% IV CD45-PE^+^ human cells in blood.

### Isolation of human ILCs from MISTRG mice and from human blood

Lung, liver, spleen, and bone marrow harvested from MISTRG mice were processed as described in (31). Briefly, small lung pieces were digested at 37°C for 1 hour in RPMI 1640 with 5% FCS, 0.2 mg/mL collagenase IV (Sigma) and 0.02 mg/mL DNAse I (Sigma). Digested lung pieces were mashed using a syringe plunger to mechanically dissociate the cells and passed through a 70 µm filter. Lung lymphocytes were then further purified by density gradient centrifugation with Lymphoprep (Fisher Scientific). Like the lungs, crushed liver pieces were digested at 37°C for 1 hour in digestion media (see above) and then washed with RPMI 1640/5% FCS. To remove hepatocytes, digested livers were centrifuged at 300 rpm for 3 minutes at 4°C. Then, leukocytes in the supernatant were pelleted by centrifugation (1,700 rpm for 10 minutes at 4°C) before further purification by density gradient centrifugation with 27.5% Optiprep (Abbott Rapid Diagnostics). Spleen were mechanically dissociated with a syringe plunger before passing through a 70 µm filter. For bone marrow, hind legs harvested from MISTRG mice were cleaned, cut at both ends, and bone marrow cells flushed from the bone with a syringe and 25G needle. Cells isolated from all four organs were treated with red blood cell lysis buffer (from Karolinska University Hospital), washed with RPMI 1640, and counted prior to staining for flow cytometry. Blood was obtained from HSPC-engrafted MISTRG mice by cardiac puncture and red blood cell lysis performed before staining cells for flow cytometry. Density gradient centrifugation was used to isolate peripheral blood mononuclear cells from human cord blood and buffy coats. The CD34^-^ fraction from cord blood (after immunomagnetic selection) was used for flow cytometry of ILCs.

### Flow cytometry of human ILCs

Surface staining of immune cells isolated from human blood or from HSPC-engrafted MISTRG mice was performed essentially as previously described (31). Briefly, suspensions of single cells were incubated with the fluorochrome- or biotin-conjugated antibodies listed in **Supplementary Table 1** in 100 μl FACS buffer (PBS/2% FCS) for 1 hour at room temperature. After washing, cells were incubated with streptavidin-Brilliant Violet 711 (BD Biosciences) for 30 minutes on ice. Then, surface-stained cells were incubated with fixable viability dye-eFluor506 (eBioscience) for 15 minutes on ice. For detection of Ki67 and transcription factors, cells were fixed and permeabilized with the Foxp3/Transcription Factor Staining kit (eBioscience) after surface and viability staining. Fixed and permeabilized cells were then stained with antibodies against transcription factors or Ki67 as well as matched isotype control antibodies. For cell cycle analysis, Ki67-stained cells were incubated with 20 μl propidium iodide (PI) (eBioscience) per 5 x 10^6^ cells for 30 minutes at room temperature in 100 μl Cytoperm buffer (BD Biosciences) containing RNase A. Intracellular staining for IFNγ was performed with the Cytofix/Cytoperm kit (BD Biosciences) as described (31) after 6 hours of stimulation with phorbol 12-myristate 13-acetate (PMA) (Sigma) and ionomycin (Sigma). After cell acquisition on a LSR II Fortessa flow cytometer (BD Biosciences), FlowJoV10 software was used for data analysis. ILCs were gated as viable human CD45^+^CD127^+^CD94^-^CD3^-^TCRαβ^-^ cells that were negative for Lineage (Lin) markers (CD11c, CD14, CD19, CD123, FceRI) as well as lacking human CD34 and mouse CD45 as indicated.

### Single-cell RNA-sequencing of ILCs

Cells for single-cell RNA-sequencing were isolated from the spleens of two months-old irradiated and HSPC-engrafted MISTRG mice after intravascular cell labeling was performed with IV-injected anti-CD45-PE antibody (see above). To obtain sufficient cells, spleen cells were pooled from ten MISTRG mice, engrafted with three pooled batches of human CD34^+^ cells to reduce the impact of HSPC donor variability. ILCs were then purified as live CD45^+^CD127^+^CD94^-^CD3^-^TCRαβ^-^Lin^-^CD34^-^ lymphocytes and divided into intravascular (IV-CD45-PE^+^) and extravascular (IV-CD45-PE^-^) ILCs. Lin markers included CD11c, CD14, CD19, CD123, FceRI. Single-cell libraries were prepared using the 10x Genomics Single Cell 3’ Library v2 kit according to the manufacturer’s instructions. Libraries were sequenced on a Nextseq 550 (Illumina) and mapped to the human GRCh38 reference genome using the Cell Ranger 3.0.1 pipeline (10x Genomics). Both extravascular and intravascular samples were analyzed using Seurat 4.1.1, with cut-offs applied for genes expressed in a minimum of 3 cells, and cells expressing a minimum of 200 genes. The samples were then merged into a single Seurat object using the merge function. Further quality control selected cells with > 100 genes, < 4,000 genes, and < 5 percent mitochondrial genes, using the subset function on the merged dataset. Log normalization to 10,000 counts was next applied to the remaining 5,853 cells. The FindVariableFeatures function was applied to select 2,000 highly variable genes, followed by ScaleData, which was then used to produce a PCA and neighborhood graph (10 principal components). Louvain clustering was applied with the FindClusters function with resolution 0.45, resulting in 11 clusters (**Supplementary Figure 5A**). The FindAllMarkers function (min.pct=0.25, logfc.threshold=0.25) function was applied to identify marker genes for each cluster, pct.1 represents the fraction of cells expressing the gene in the specified cluster being tested, while pct.2 represents the remaining cells. To better represent the main ILC populations we applied the subset function to extract clusters containing ILCs (Clusters 0-5), resulting in 4,936 remaining cells (2,704 vascular and 2,232 tissue cells). PCA, neighborhood graph (10 principal components), and Louvain clustering (resolution 0.44) was reapplied to the new Seurat object, resulting in 8 clusters. This was the Seurat object used for all further analysis. Uniform Manifold Approximation and Projection (UMAP) embedding was produced using 10 principal components (**Figure 7B**). The FindAllMarkers function (min.pct=0.25, logfc.threshold=0.25) function was applied to identify marker genes for each cluster in the final object. Violin and feature plots were produced using the VlnPlot and FeaturePlot function, respectively. Cell cycle analysis of ILC clusters was performed with the CellCycleScoring function. To identify biological processes over-represented within the gene signature of *MKI67*-ILCs, WebGestalt (http://webgestalt.org/) was employed using default parameters.

### Statistical analysis

For statistical comparisons between two groups Student’s *t* test was used. One-way ANOVA was employed to determine statistical significance between multiple groups. ANOVA *post hoc* testing was done with Tukey’ Multiple Comparison Test. Results were considered statistically significant with *α*= 0.05. Mean and standard error of the mean (SEM) are shown in the figures. The number of biological replicates and independent experiments are stated in the figure legends. GraphPad Prism 8 was used to perform statistical analysis and create graphs.

### Cartoons to illustrate experimental outlines

**Figure 1:**
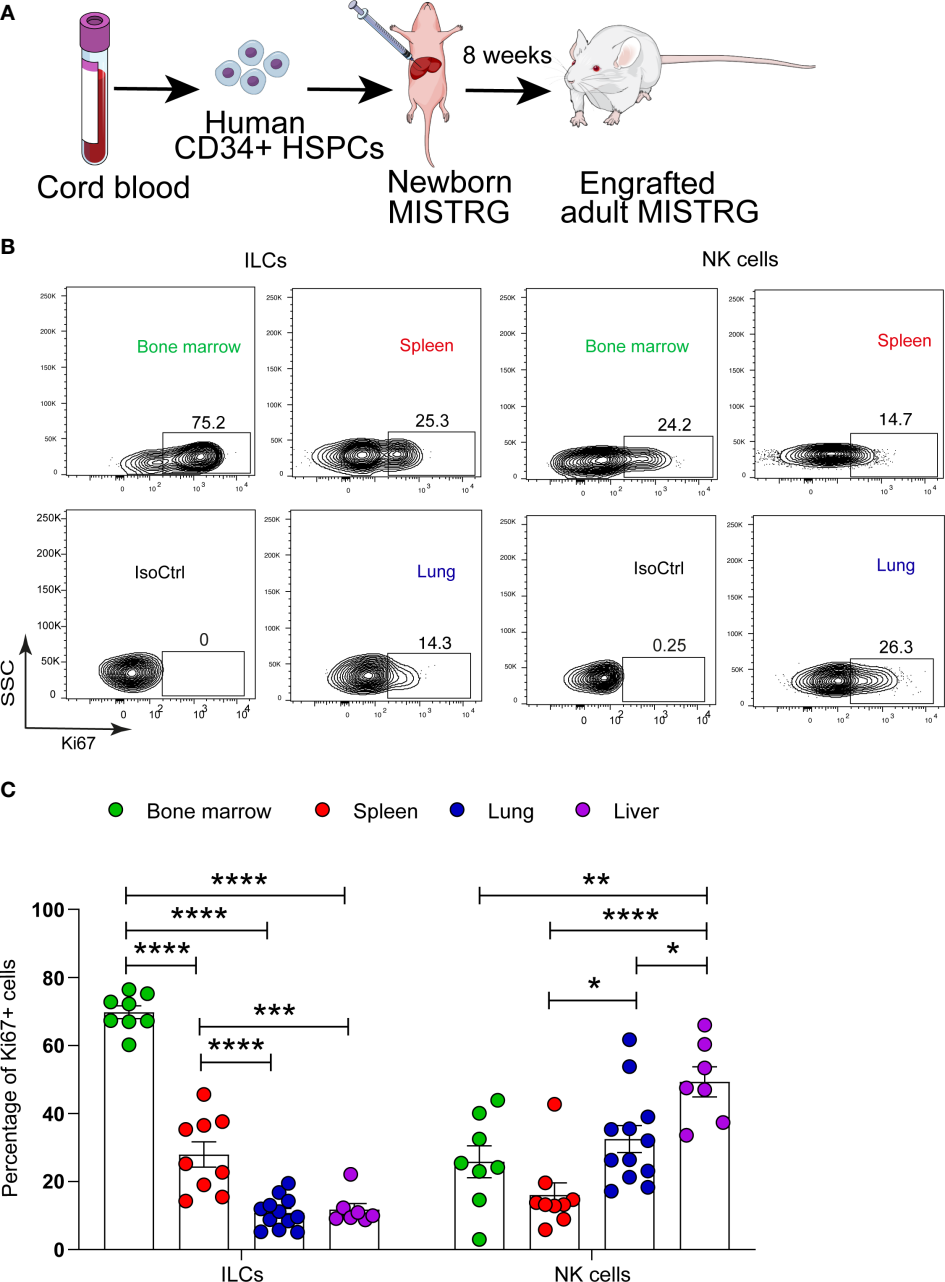
Proliferative status of human ILCs in lymphoid and non-lymphoid organs of HSPC-engrafted MISTRG mice. **(A)** Transplantation of newborn MISTRG mice with human CD34^+^ cord blood HSPCs. **(B, C)** Intracellular Ki67 expression by human ILCs and NK cells as determined by flow cytometry. Isotype control (IsoCtrl) staining is shown for the lung. ILCs were gated as human CD45^+^CD127^+^CD94^-^CD3^-^TCRαβ^-^Lin^-^ cells and NK cells were gated as human CD45^+^CD127^-^CD94^+^CD3^-^TCRαβ^-^Lin^-^ cells as in **Supplementary Figure 1**. For bone marrow analysis, ILCs and NK cells were also gated as CD34^-^ cells. Frequencies of Ki67^+^ ILCs and NK cells in the indicated organs of HSPC-engrafted MISTRG mice (n = 7-12) are shown in **(C)**. *, P <0.05; **, *P <*0.01; ***, P <0.001; ****, *P <*0.0001 by one-way ANOVA, Tukey’s post-test. Data are represented as mean ± SEM. Data are from two independent experiments using MISTRG mice engrafted with different pools of HSPCs. (A) was created with Mind the Graph.

**Figure 2:**
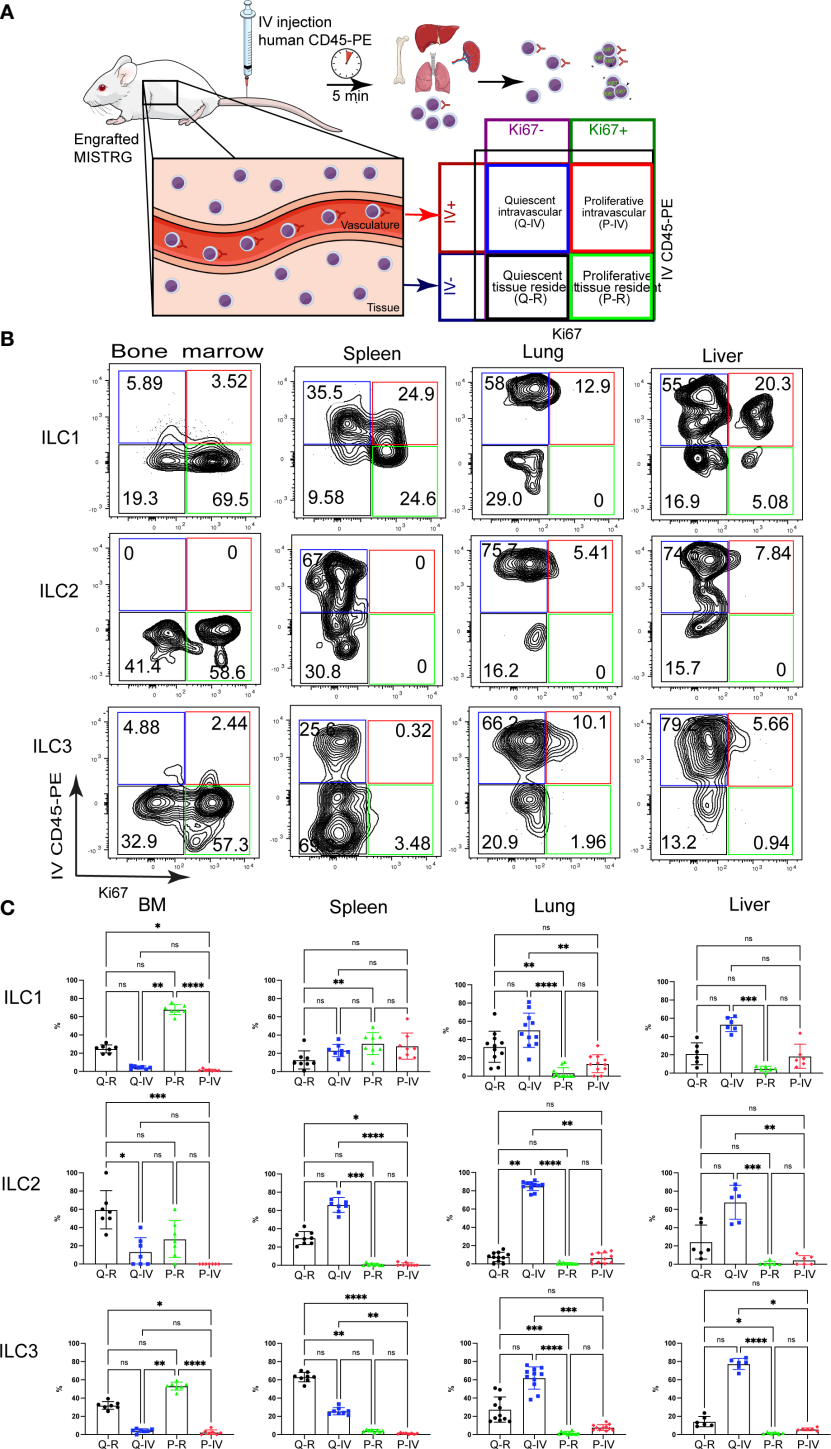
Spatial distribution of quiescent and proliferative ILCs within vascular and tissue compartments. **(A)** Experimental outline. Intracellular Ki67 staining of human ILCs was performed in HSPC-engrafted MISTRG mice after (IV) injection of anti-human CD45-PE antibody to label intravascular human hematopoietic cells. Human cells in the organ vasculature are stained by the IV-injected anti-CD45 antibody (IV CD45-PE), whereas cells residing in the tissue are not stained. Following intravascular labeling, cells were surface-stained *ex vivo* and stained intracellularly with an antibody against Ki67. Then quiescent (Ki67^-^) and proliferative (Ki67^+^) as well as intravascular (IVCD45-PE^+^) and extravascular ILCs within tissue (IVCD45-PE^-^) were distinguished by flow cytometry. **(B)** Flow cytometry analysis of intravascular versus extravascular and quiescent versus proliferative CD117^-^CRTH2^-^ ILC1s, CRTH2^+^ ILC2s, and CD117^+^ ILC3s in the indicated organs of HSPC-engrafted MISTRG mice. ILCs were gated as human CD45^+^CD127^+^CD94^-^CD3^-^TCRαβ^-^Lin^-^ cells as in **Supplementary Figure 1** and as CD34^-^ cells. **(C)** Frequencies of quiescent tissue-resident (Q-R), quiescent intravascular (Q-IV), proliferative tissue-resident (P-R), and proliferative intravascular (P-IV) human ILC1s, ILC2s, and ILC3s in the indicated organs (n = 6-11) as determined in (B). n.s., not significant; *, P <0.05; **, *P <*0.01; ***, P <0.001; ****, *P <*0.0001 by one-way ANOVA, Tukey’s post-test. Data represent mean ± SEM and are representative of two independent experiments using MISTRG mice engrafted with different pools of HSPCs. (A) was created with Mind the Graph.

The cartoons in **Figures 1A**, **2A**, **7A**, and **S6** were prepared using images from Mind the Graph (http://mindthegraph.com).

## Results

### Proliferative status of human ILCs in lymphoid and non-lymphoid organs

We first asked in which organs ILC proliferation takes place. To investigate the link between human ILC localization and proliferation, we used MISTRG mice transplanted with human CD34^+^ HSPCs (**Figure 1A**) as in our previous studies (27, 31, 33). MISTRG mice were not pre-conditioned by irradiation before HSPC transplantation. ILCs derived from human HSPCs were gated as CD45^+^CD127^+^CD94^-^ cells that lacked T cell markers (CD3, TCRαβ) and other Lin markers (CD11c, CD14, CD19, CD123, FceRI) (**Supplementary Figure 1**). Then the cell division status of human ILCs and NK cells (CD45^+^CD127^-^CD94^+^CD3^-^TCRαβ^-^Lin^-^) was determined by intracellular staining for the proliferation marker Ki67 in hematopoietic (bone marrow), lymphoid (spleen), and non-lymphoid organs (lung, liver). Flow cytometry analysis revealed a high frequency of Ki67^+^ ILCs in bone marrow, while in peripheral organs the highest frequency of proliferating ILCs was found in the spleen (**Figures 1B, C**). In contrast, proliferating NK cells (**Figures 1B, C**) and T cells (**Supplementary Figures 2A, B**) were mostly located in non-lymphoid organs (lung and liver) as well as in the bone marrow. These results indicate that ILCs have an organ-specific proliferative hierarchy that is distinct from that of NK cells and T lymphocytes.

### Spatial distribution of quiescent and proliferative ILCs within vascular and tissue compartments

The above findings suggested that human ILC proliferation may be spatially compartmentalized and prompted us to determine the localization of proliferating ILCs within vascular and tissue compartments. To distinguish vascular from tissue-resident Ki67^+^ ILCs, we employed intravascular labeling of human CD45^+^ hematopoietic cells by the IV injection of a PE-conjugated anti-human CD45 antibody (**Figure 2A**), as we did previously (31, 33). This experimental approach allowed us to distinguish four different ILC populations (**Figure 2A**): (i) quiescent tissue-resident (Q-R) ILCs (Ki67^-^ IVCD45-PE^-^), (ii) quiescent intravascular (Q-IV) ILCs (Ki67^-^ IVCD45-PE^+^), (iii) proliferative tissue-resident (P-R) ILCs (Ki67^+^ IVCD45-PE^-^), and (iv) proliferative intravascular (P-IV) ILCs (Ki67^+^ IVCD45-PE^+^). The distribution of these four populations differed among ILC subsets (CD117^-^CRTH2^-^ ILC1s, CRTH2^+^ ILC2s, CD117^+^CRTH2^-^ ILCPs/ILC3s) in different organs (**Figures 2B, C**). ILC2s were mostly quiescent and intravascular in spleen, lung, and liver (**Figures 2B, C**). NK cells (**Supplementary Figures 3A, B**) and T lymphocytes (**Supplementary Figure 3C, D**) showed a similar pattern, but with more proliferating cells within the lung and liver vasculature than ILC2s. ILC3s and CD117^+^ ILCPs were largely quiescent in spleen, lung, and liver, whereas in the bone marrow they were proliferative and tissue-resident (**Figures 2B, C**). In the spleen ILC3s and CD117^+^ ILCPs were located preferentially within the tissue compartment, whereas they mostly had an intravascular localization in the lung and liver (**Figures 2B, C**). CD117^-^CRTH2^-^ ILC1s on the other hand were more proliferative than the other ILC subsets, especially in the spleen (**Figures 2B, C**). Moreover, Ki67^+^ ILC1s were present in the local vasculature of peripheral organs (spleen, lung, liver), whereas in the bone marrow they mostly resided in the extravascular space (**Figures 2B, C**). Combined, these data suggest that human ILC subsets differ in their proliferative state and spatial compartmentalization within organs.

### CD117^-^CRTH2^-^CD45RA^+^ ILCs in the spleen are highly proliferative

**Figure 3:**
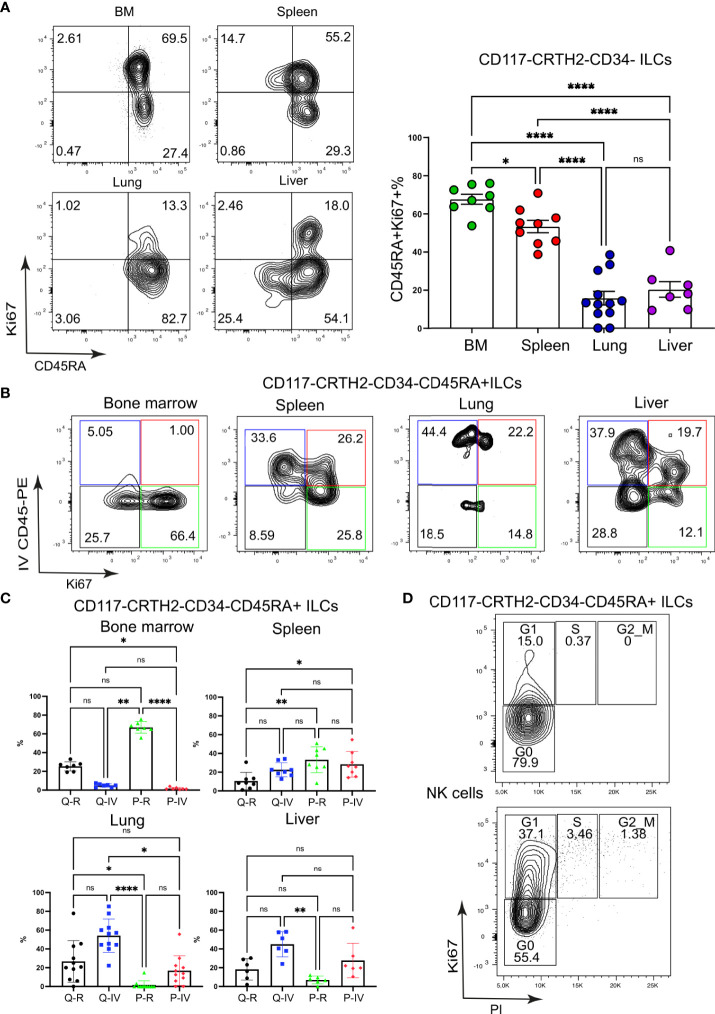
CD117^-^CRTH2^-^CD45RA^+^ ILCs in the spleen are highly proliferative. **(A)** Frequencies of proliferating CD117^-^CRTH2^-^CD34^-^ ILCs according to CD45RA surface expression in various organs of HSPC-engrafted MISTRG mice (n = 7-12). ILCs were gated as human CD45^+^CD127^+^CD94^-^CD3^-^TCRαβ^-^Lin^-^ cells as in **Supplementary Figure 1** and then gated as CD117^-^CRTH2^-^CD34^-^ ILCs. **(B)** Flow cytometry analysis of intravascular versus extravascular and quiescent versus proliferative CD117^-^CRTH2^-^CD34^-^ ILCs in the indicated organs of HSPC-engrafted MISTRG mice. ILCs were gated as human CD45^+^CD127^+^CD94^-^CD3^-^TCRαβ^-^Lin^-^ cells as in **Supplementary Figure 1** and then gated as CD117^-^CRTH2^-^CD34^-^ ILCs. **(C)** Frequencies of quiescent tissue-resident (Q-R), quiescent intravascular (Q-IV), proliferative tissue-resident (P-R), and proliferative intravascular (P-IV) CD117^-^CRTH2^-^CD34^-^ ILCs in the indicated organs (n = 6-11) as determined in **(B)**. **(D)** Cell cycle status of CD117^-^CRTH2^-^CD45RA^+^ ILCs and NK cells from the spleen of HSPC-engrafted MISTRG mice (n = 4). n.s., not significant; *, P <0.05; **, *P <*0.01; ***, P <0.001; ****, *P <*0.0001 by one-way ANOVA, Tukey’s post-test. Data represent mean ± SEM and are representative of two independent experiments using MISTRG mice engrafted with different pools of HSPCs. **(A)** was created with Mind the Graph.

Having discovered discrete subsets of Ki67^+^ ILCs, we further examined the proliferation of ILCs with an ILC1 surface phenotype (CD117^-^CRTH2^-^). For this purpose, we divided ILC1s into two subsets based on surface expression of CD45RA, a marker for immature ILCs (24, 34, 35), although it is also uniformly expressed by mature NK cells. Ki67^+^ cells among CD117^-^CRTH2^-^ ILCs mostly expressed CD45RA on their cell surface in all organs examined (**Figure 3A**). Moreover, there was a large population of CD117^-^CRTH2^-^CD45RA^+^ ILCs in the spleen that was Ki67^+^ and present in both the intravascular and extravascular compartment of the spleen (**Figures 3B, C**). Intravascular Ki67^+^ CD45RA^+^ ILC1s were also present in lung and liver, whereas proliferating CD45RA^+^ ILC1s were predominantly extravascular in the bone marrow (**Figures 3B, C**). Further flow cytometric analysis revealed that proliferative CD117^-^CRTH2^-^CD45RA^+^ ILCs in the spleen were mostly in G1 phase of the cell cycle (**Figure 3D**). We conclude that proliferative CD117^-^CRTH2^-^ ILCs have an immature surface phenotype and mostly reside within both the vascular and tissue compartment of the spleen.

### Proliferative CD117^-^CRTH2^-^CD45RA^+^ ILCs express the transcription factor TCF-1

**Figure 4:**
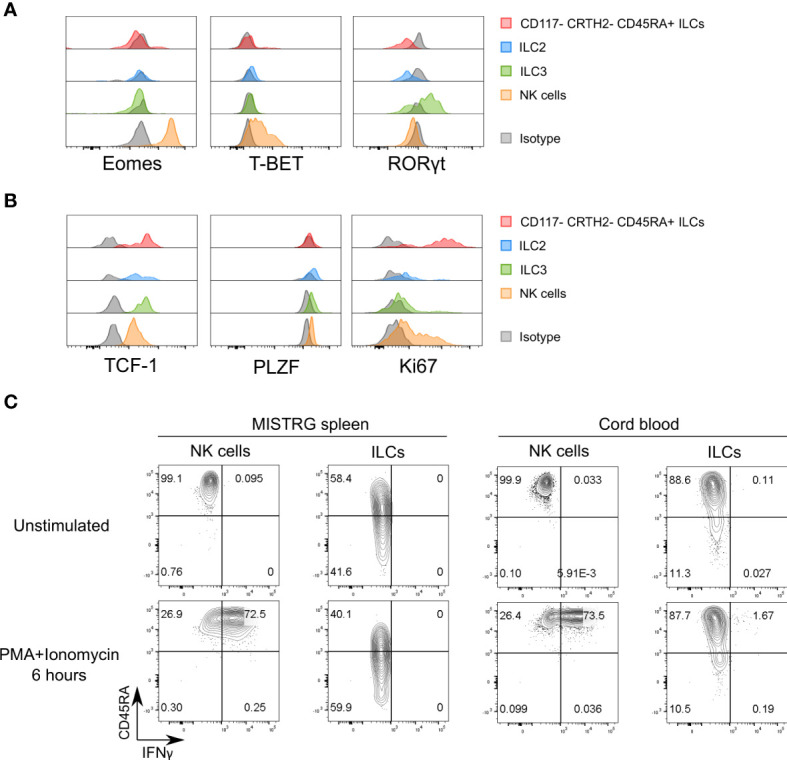
Proliferative CD117^-^CRTH2^-^CD45RA^+^ ILCs express the transcription factor TCF-1. **(A)** Intracellular expression of signature transcription factors (Eomes, T-BET, RORγt) in human CD117^-^CRTH2^-^CD45RA^+^ ILCs, CRTH2^+^ ILC2s, CD117^+^CRTH2^-^ ILC3s, and NK cells isolated from the spleen of HSPC-engrafted MISTRG mice (n = 5). Matched isotype antibodies were used as controls. **(B)** Intracellular expression of transcription factors (TCF-1, PLZF) and Ki67 in human CD117^-^CRTH2^-^CD45RA^+^ ILCs, CRTH2^+^ ILC2s, CD117^+^CRTH2^-^ ILC3s, and NK cells isolated from the spleen of HSPC-engrafted MISTRG mice (n = 6). Matched isotype antibodies were used as controls. **(C)** Intracellular IFNγ expression by human ILCs and NK cells in the spleen of HSPC-engrafted MISTRG mice and in umbilical cord blood (n = 4). Cells were either unstimulated or stimulated with PMA and ionomycin for 6 hours. ILCs were gated as human CD45^+^CD127^+^CD94^-^CD3^-^TCRαβ^-^Lin^-^ cells and NK cells were gated as human CD45^+^CD127^-^CD94^+^CD3^-^TCRαβ^-^Lin^-^ cells as in **Supplementary Figure 1**. ILC subsets and NK cells were also gated as CD34^-^ cells. Data represent mean ± SEM and are representative of two **(B, C)** or three (A) independent experiments.

To further elucidate the features of proliferating CD117^-^CRTH2^-^CD45RA^+^ ILCs, we examined the expression of transcription factors that define ILC lineages. As expected, CD117^-^CRTH2^-^CD45RA^+^ ILCs from the spleen of HSPC-engrafted MISTRG mice lacked expression of EOMES and RORγt (**Figure 4A**). In addition, the majority of splenic CD117^-^CRTH2^-^CD45RA^+^ ILCs did not express intracellular T-BET protein, the signature transcription factor for ILC1s, whereas NK cells were uniformly T-BET^+^ (**Figure 4A**). The observation that most CD117^-^CRTH2^-^ ILCs were Ki67^+^ but lacked intracellular T-BET protein, suggested that CD117^-^CRTH2^-^ ILCs contained proliferating immature ILCs. To further explore this possibility, we determined expression of the transcription factor TCF-1 that is associated with proliferative potential (11, 24, 36, 37). We also assessed the expression of PLZF, another transcription factor associated with ILC development (38–41). CD117^-^CRTH2 CD45RA^+^ ILCs expressed high amounts of intracellular TCF-1 protein, but not PLZF, which was expressed by the other ILC subsets including NK cells (**Figure 4B**). CD117^+^CRTH2^-^ ILCPs/ILC3s also highly expressed TCF-1 but with only few Ki67^+^ cells, in contrast to CD117^-^CRTH2^-^CD45RA^+^ ILCs that were TCF-1^hi^Ki67^+^ (**Figure 4B**). Therefore, two populations of ILCs with high TCF-1 expression could be distinguished, namely proliferative CD117^-^CRTH2^-^CD45RA^+^ ILCs and quiescent CD117^+^ ILCPs/ILC3s. The lack of T-BET expression indicated that splenic CD117^-^CRTH2^-^CD45RA^+^ ILCs do not represent *bona-fide* ILC1s. To further corroborate this notion, we examined production of IFNγ, the signature cytokine produced by ILC1s. As expected, most human NK cells expressed intracellular IFNγ protein in response to stimulation with PMA and ionomycin *in vitro* (**Figure 4C**). In contrast, neither human ILCs from the spleen of HSPC-engrafted MISTRG mice nor human ILCs from umbilical cord blood produced IFNγ after stimulation (**Figure 4C**). In summary, we found that CD117^-^CRTH2^-^ ILCs in the spleen contain a proliferative population that expressed TCF-1 and had features of functionally immature ILCs.

### Proliferative CD117^-^CRTH2^-^CD45RA^+^ ILCs are present in the circulation and in human umbilical cord blood

**Figure 5:**
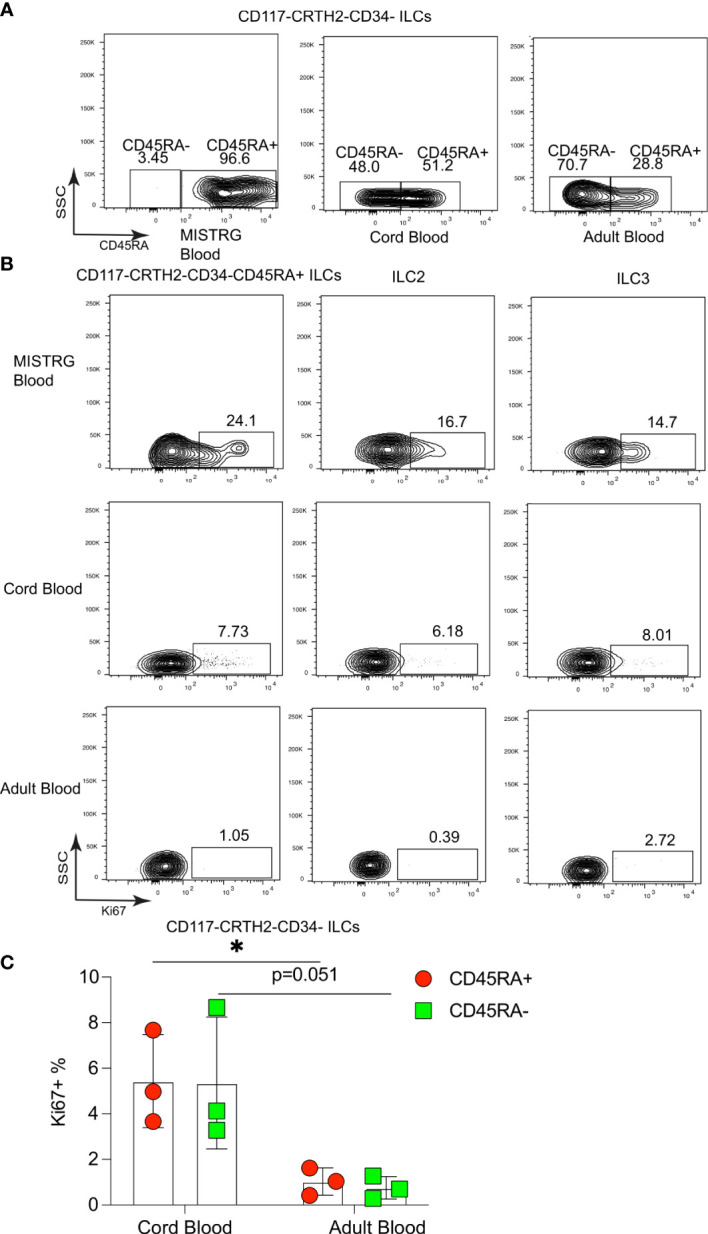
Proliferative CD117^-^CRTH2^-^CD45RA^+^ ILCs are present in the circulation and in human umbilical cord blood. **(A)** CD45RA surface expression by CD117^-^CRTH2^-^ ILCs from the blood of HSPC-engrafted MISTRG mice, human umbilical cord blood, and human adult blood (n = 3). **(B)** Flow cytometry analysis of intracellular Ki67 expression by human CD117^-^CRTH2^-^CD34^-^CD45RA^+^ ILCs, CRTH2^+^ ILC2s, and CD117^+^CRTH2^-^ ILC3s from the blood of HSPC-engrafted MISTRG mice as well as from human cord blood and human adult blood (n = 3). ILCs were gated as human CD45^+^CD127^+^CD94^-^CD3^-^TCRαβ^-^Lin^-^ cells as in **Supplementary Figure 1**. **(C)** Frequencies of Ki67^+^ human CD117^-^CRTH2^-^CD34^-^CD45RA^+^ and CD117^-^CRTH2^-^CD34^-^CD45RA^-^ ILCs in human cord blood and adult blood (n = 3). *, P <0.05 by Student’s *t* test. Data represent mean ± SEM and are from three independent experiments.

We next asked whether proliferative CD117^-^CRTH2^-^CD45RA^+^ ILCs resided in local vascular compartments and/or circulated between organs. The latter predicted that Ki67^+^ CD117^-^CRTH2^-^CD45RA^+^ ILCs were present in the systemic circulation. Consistent with this prediction, we found that circulating human CD117^-^CRTH2^-^ ILCs in HSPC-engrafted MISTRG mice were mostly CD45RA^+^ (**Figure 5A**). Umbilical cord blood also contained a substantial proportion of CD117^-^CRTH2^-^CD45RA^+^ ILCs, whereas CD117^-^CRTH2^-^CD45RA^+^ ILCs were present in adult blood at a lower frequency (**Figure 5A**). Furthermore, we detected CD117^-^CRTH2^-^CD45RA^+^ ILCs in the blood of HSPC-engrafted MISTRG mice that were Ki67^+^ (**Figure 5B**). Ki67^+^ ILC2s and ILC3s/CD117+ ILCPs were also present in the blood of HSPC-engrafted MISTRG mice (**Figure 5B**), despite being less abundant in the local vasculature of the spleen, lung, and liver than Ki67^+^ CD45RA^+^ ILC1s (**Figure 2C** and **3C**). This suggested that Ki67^+^ ILC2s and ILC3s/CD117+ ILCPs mostly recirculate through organs, whereas Ki67^+^ CD117^-^CRTH2^-^CD45RA^+^ ILCs may marginate and accumulate in the local vasculature, especially in the spleen. We next investigated whether proliferative CD117^-^CRTH2^-^CD45RA^+^ ILCs could also be detected in human blood. Ki67^+^ CD45RA^+^ ILC1s, as well as Ki67^+^ ILC2s and ILC3s/CD117+ ILCPs were rare in the blood of adult humans, consistent with a previous report (22), but more prevalent in umbilical cord blood, although less frequent than in the blood of HSPC-engrafted MISTRG mice (**Figures 5B, C**). Taken together, these findings indicate that CD117^-^CRTH2^-^CD45RA^+^ ILCs distribute *via* the vascular system and that they represent a conserved population in humans during early life.

### CD117^-^CRTH2^-^CD45RA^+^ ILCs appear early during ontogeny

**Figure 6:**
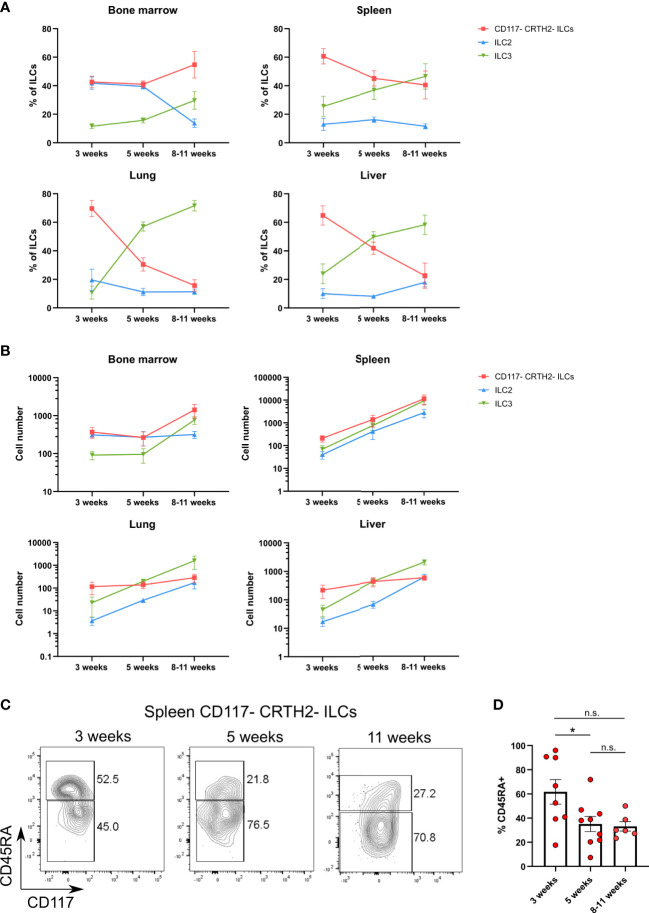
CD117^-^CRTH2^-^CD45RA^+^ ILCs appear early during ontogeny in HSPC-engrafted MISTRG mice. **(A, B)** Frequencies **(A)** and numbers **(B)** of human CD117^-^CRTH2^-^ ILCs, CRTH2^+^ ILC2s, and CD117^+^CRTH2^-^ ILC3s in bone marrow, spleen, lung, and liver at 3 weeks, 5 weeks, and 8-11 weeks after transplantation of MISTRG mice with human CD34^+^ HSPCs (n = 6-9 per time-point). ILCs were gated as human CD45^+^CD127^+^CD94^-^CD3^-^TCRαβ^-^Lin^-^ cells as in **Supplementary Figure 1** and as CD34^-^ cells. **(C)** Flow cytometry analysis of human CD117^-^CRTH2^-^ ILCs in the spleen of MISTRG mice at 3, 5, and 11 weeks after transplantation with human CD34^+^ HSPCs (n = 6-9 per time-point). ILCs were gated as human CD45^+^CD127^+^CD94^-^CD3^-^TCRαβ^-^Lin^-^ cells as in **Supplementary Figure 1** and as CD34^-^ cells. **(D)** Frequency of CD45RA^+^ cells among human CD117^-^CRTH2^-^ ILCs in the spleen of MISTRG mice at 3, 5, and 11 weeks after transplantation with human CD34^+^ HSPCs (n = 6-9 per time-point). n.s., not significant; *, P <0.05 by one-way ANOVA, Tukey’s post-test. Data represent mean ± SEM and are from two independent experiments.

Their presence during the neonatal period suggested that CD117^-^CRTH2^-^CD45RA^+^ ILCs represented circulating immature cells that may seed various organs. To further explore the ontogeny of CD117^-^CRTH2^-^CD45RA^+^ ILCs, we examined the kinetics of human ILC reconstitution in MISTRG mice after transplantation with CD34^+^ HSPCs. CRTH2^-^CD117^-^ ILCs were present in all organs including the spleen, lung, and liver (**Figures 6A, B**). At 3 weeks post-transplantation, CD117^-^CRTH2^-^ ILCs were the prevalent ILC subset in peripheral organs (spleen, liver, lung) when compared to ILC2s and ILC3s (**Figure 6A**). These data support the notion that CD117^-^CRTH2^-^ ILCs appeared early before other ILC subsets during development. In the spleen, the frequency of CD45RA^+^ cells within CD117^-^CRTH2^-^ ILCs decreased over time (**Figures 6C, D**), indicating a potential conversion of CD45RA^+^ into CD45RA^-^ CD117^-^CRTH2^-^ ILCs.

Next, we investigated the developmental niche occupied by CD117^-^CRTH2^-^CD45RA^+^ ILCs in different organs of HSPC-engrafted MISTRG mice. We found that the frequency of CD117^-^CRTH2^-^CD45RA^+^ ILCs was the same whether MISTRG mice were irradiated or not before transplantation with human CD34^+^ HSPCs (**Supplementary Figures 4A, B**). This result suggested that clearing of their anatomical niche by irradiation is not required for the development of CD117^-^CRTH2^-^CD45RA^+^ ILCs in MISTRG mice. These results are in line with human data showing that reconstitution of CD117^-^CRTH2^-^ ILC1s after hematopoietic stem cell transplantation occurs in the absence of myeloablation (42). Collectively, these data demonstrate that CD117^-^CRTH2^-^CD45RA^+^ ILCs appear early during development and inhabit a radio-insensitive developmental niche. These features indicate that CD117^-^CRTH2^-^CD45RA^+^ ILCs may contribute to the expanding ILC compartment in early life.

### Heterogeneity of vascular and tissue ILCs in the spleen defined by single-cell RNA-sequencing

**Figure 7:**
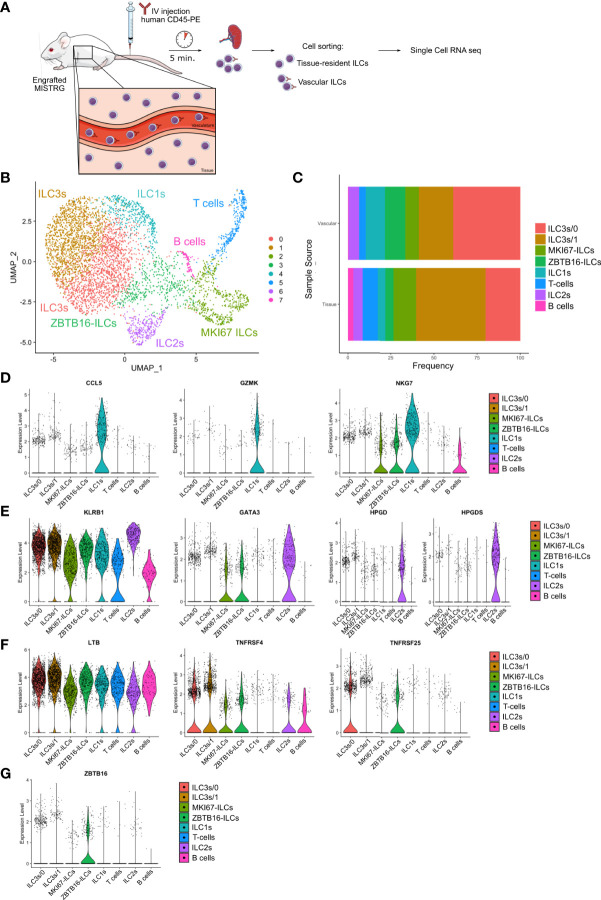
Heterogeneity of vascular and tissue ILCs in the spleen defined by single-cell RNA-sequencing. **(A)** Experimental outline. Human CD127^+^CD94^-^ ILCs were purified from both the intravascular and the extravascular (tissue) compartment of the spleen of HSPC-engrafted MISTRG mice and subjected to single-cell RNA-sequencing. **(B)** UMAP depicting human ILC clusters (4,935 cells) in the spleen of HSPC-engrafted MISTRG mice. **(C)** Frequency of ILC clusters within the vascular versus the tissue compartment of the spleen. **(D–G)** Violin plots showing expression of selected genes characteristic of ILC1 **(D)**, ILC2 **(E)**, ILC3 **(F)**, and *ZBTB16*-ILC **(G)** clusters. Data are from one single-cell RNA-sequencing experiment with spleen cells pooled from ten MISTRG mice engrafted with three pooled batches of human CD34^+^ cells. **(A)** was created with Mind the Graph.

To further define the heterogeneity of human ILCs located in the intra- and extravascular compartments of the spleen, we employed single-cell RNA-sequencing using the 10x Genomics platform (**Figure 7A**). For this purpose, human ILCs were purified from the spleens of HSPC-engrafted MISTRG mice by cell sorting. Before spleen harvest, intravascular cell labeling with IV CD45-PE antibody was performed to isolate ILCs from the intravascular (IV CD45-PE^+^) and extravascular space (IV CD45-PE^-^) for single-cell RNA-sequencing. 11 transcriptionally different clusters could be distinguished after unsupervised clustering of cells by UMAP (**Supplementary Figure 5A**). After removal of minor contaminating clusters consisting of myeloid cells and red blood cells, 8 clusters consisting of 4,936 lymphocytes remained for further analysis (**Figure 7B**). Cluster 7 corresponded to B lymphocytes (*JCHAIN, CD79A, HLA-DPA1*) and cluster 5 was identified as T lymphocytes or T cell progenitors (*CD3D, CD3G, TRBC1, RAG1, CD1B, CD1E, CD8B, LEF1*) that lacked TCRαβ and CD3 surface expression (**Supplementary Table 2**).

We then focused our analysis on the six remaining ILC clusters. Cells in cluster 4 mostly had an intravascular localization (**Figure 7C**) and were identified as ILC1s (and/or possibly CD127^+^CD94^-^ NK cells) based on genes expressed by ILC1s in human spleen (23), such lytic granule molecules (*GZMK, GNLY, NKG7*) and the chemokine *CCL5* (**Figure 7D** and **Supplementary Table 2**). Cluster 6 expressed genes characteristic of ILC2s, such as the signature transcription factor *GATA3*, transcripts for the ILC2 surface markers *KLRB1* (encoding CD161) and *KLRG1*, as well as the ILC2 signature genes *HPGD* and *HPGDS* (**Figure 7E** and **Supplementary Table 2**). ILC2s were present in both the vascular and tissue compartment of the spleen (**Figure 7C**).

Two separate clusters of ILC3s were present in the spleen (**Figure 7B**) that differed in their anatomical location. ILC3s of cluster 0 predominantly occupied the intravascular compartment of the spleen (**Figure 7C**). Consistent with their ILC3 identity, cells in cluster 0 were characterized by transcripts that are expressed by ILC3s in human spleen and other secondary lymphoid organs, such as *TNFRSF25* (encoding DR3), *TYROBP*, *NFKBIA*, *ZFP36* (23, 34, 43). In addition, ILC3s in cluster 0 expressed genes involved in cell migration, such as the adhesion molecules *ITGB1* and *CD44*, as well as genes encoding S100A proteins (*S100A4, S100A6, S100A10*) (**Figure 7F** and **Supplementary Table 2**). Their gene signature suggested that intravascular ILC3s actively migrate into the extravascular space of the spleen. The second cluster (cluster 1) preferentially resided within the tissue compartment of the spleen (**Figure 7C**). ILC3s in cluster 1 expressed the transcription factor *ID2*, as well as the ILC3 signatures genes *LTB* and *TNFRSF4* (encoding OX40) that are involved in lymphoid tissue organogenesis and interaction of ILC3s with other cells (**Figure 7F** and **Supplementary Table 2**). Cluster 1 cells transcribed other genes that are expressed by human ILC3s in secondary lymphoid organs, such as *B2M, HLA-B*, *HLA-C*, *TNFRSF18* (encoding GITR), *TYROBP*, *IFITM1*, *IFITM2* (26, 34, 43).

ILCs in cluster 3 were characterized by expression of *ZBTB16* (**Figure 7G** **Supplementary Table 2**), encoding the transcription factor PLZF that is expressed by all human ILC subsets (34, 44, 45) and required for ILC development in mice (39, 46, 47). Otherwise, *ZBTB16*-ILCs expressed few distinctive genes (**Supplementary Table 2**), suggesting that they may be less differentiated. Finally, the frequency of *ZBTB16*-ILCs was greater in the spleen vasculature than in spleen tissue (**Figure 7C**). In conclusion, we found that not only known human ILC subsets are present in the spleen of HSPC-engrafted MISTRG mice, but also a distinct population of *ZBTB16*-ILCs which may represent an intermediate ILC differentiation stage.

### Single-cell gene signature of proliferative *MKI67*-ILCs

**Figure 8:**
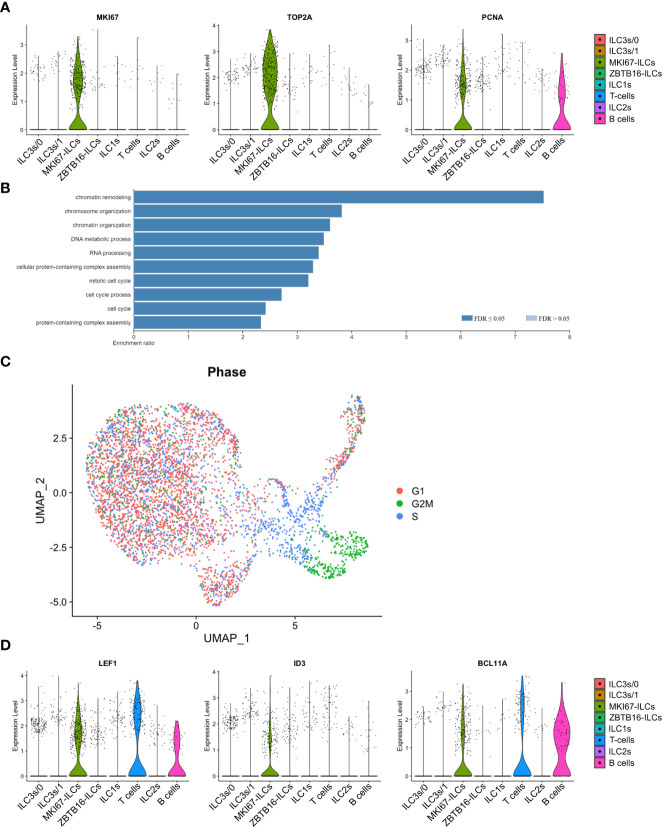
Single-cell gene signature of proliferative *MKI67*-ILCs. **(A)** Violin plots showing expression of proliferation-associated genes in the human ILC clusters from the UMAP in **Figure 7B**. **(B)** Gene Ontology over-representation analysis of genes differentially expressed by *MKI67*-ILCs. FDR, false-discovery rate. **(C)** Cell cycle scoring of ILCs superimposed on the UMAP from **Figure 7B**. **(D)** Violin plots showing expression of selected transcription factors in the human ILC clusters from the UMAP in **Figure 7B**. Data are from one single-cell RNA-sequencing experiment with spleen cells pooled from ten MISTRG mice engrafted with three pooled batches of human CD34^+^ cells.

Cluster 2 ILCs were distinguished by the expression of genes associated with cell proliferation, such as *MKI67*, *TOP2A*, *PCNA*, and *STMN1* as well as cyclin-dependent kinases (**Figure 8A** and **Supplementary Table 2**). Accordingly, gene ontology categories overrepresented in cluster 2 ILCs included “mitotic cell cycle” and “cell cycle process” (**Figure 8B**). Cell cycle scoring analysis confirmed that *MKI67*-ILCs were proliferative as most cells in this cluster were scored as in S or G2/M phase (**Figure 8C**). Furthermore *MKI67*-ILCs were present in both the intra- and extravascular compartment of the spleen (**Figure 7C**). *MKI67*-ILCs expressed low amounts of the transcription factors *EOMES*, *TBX21*, and *RORC* (**Supplementary Figures 5B, C**) and lower amounts of *GATA3* than ILC2s in cluster 6 (**Figure 7E**), suggesting that *MKI67*-ILCs did not correspond to NK cells, ILC1s, ILC2s, or ILC3s. However, *MKI67*-ILCs expressed *TCF7* (encoding TCF-1) (**Supplementary Figures 5B, C**), consistent with the TCF-1 protein expression determined by flow cytometry (**Figure 4B**). *MKI67*-ILCs also expressed several transcription factors, such as *LEF1, ID3, BCL11A*, and *BCL11B* (**Figure 8D**), that regulate the proliferative potential of lymphocytes. LEF1 is downstream of the WNT signaling pathway and is associated with the self-renewal capacity of T lymphocytes and NK cells (36). Moreover, *LEF1* has been reported to be more highly expressed by human CD117^-^CRTH2^-^ ILCs than by other ILC subsets and NK cells (34, 45). *ID3* is expressed by TCF-1^+^ CD8 T cells with stem cell-like features (48–50) and by human cord blood ILCs that are functionally immature (51). Finally, *BCL11B* is expressed by human ILC1-like cells in umbilical cord blood (52), while *BCL11A* expression is shared by CD117^-^CRTH2^-^ ILCs in human spleen and CD34^+^CD127^+^ common lymphoid progenitors from umbilical cord blood (23). In summary, these data support the notion that a distinct cluster of proliferative human ILCs resides in the spleen.

## Discussion

The function of ILCs is tightly linked to their anatomical location and, like other tissue-resident immune cells, such as macrophages, the local microenvironment imprints tissue-specific features on ILCs (19, 53). However, the proliferative hierarchy and anatomical compartmentalization of human ILCs *in vivo* has not been determined. Here, we visualized proliferating ILCs within the vascular and tissue compartments using intravascular cell labeling and single-cell RNA-sequencing in our MISTRG humanized mouse model. This allowed us to define the proliferative landscape of human ILCs within lymphoid and non-lymphoid organs (**Supplementary Figure 6**).

We found that proliferating ILCs occupied both the vascular and tissue space in various organs. Specifically, our approach allowed us to classify ILCs into four categories with distinct proliferative status and anatomical location (**Supplementary Figure 6A**). The first category corresponded to quiescent and intravascular ILCs. NK cells and ILC2s in spleen, lung, and liver as well as ILC3s in lung and liver belonged to this category. The second category of quiescent and tissue-resident ILCs was mainly represented by CD117^+^CRTH2^-^ ILC3s and ILCPs (24) in the spleen. The third category of proliferative and intravascular ILCs included lung and liver NK cells as well as the proliferative CD117^-^CRTH2^-^CD45RA^+^ ILCs that we discovered in the spleen vasculature and in the systemic circulation. CD117^-^CRTH2^-^CD45RA^+^ ILCs in the spleen also belonged to the fourth category of proliferative and tissue-resident ILCs.

The highly proliferative CD117^-^CRTH2^-^ human ILC population that we discovered had a naïve surface phenotype (CD45RA^+^) and was characterized by the absence of transcription factors associated with mature ILCs. Despite having a surface phenotype (CD117^-^CRTH2^-^) that is normally associated with ILC1s, most CD117^-^CRTH2^-^CD45RA^+^ ILCs lacked expression of the ILC1-defining transcription factor T-BET and did not produce IFNγ. Instead, this population expressed TCF-1, characteristic of lymphocytes with high proliferative capacity (54, 55). Therefore, proliferative CD117^-^CRTH2^-^ ILCs mostly represented CD45RA^+^T-BET^lo^TCF-1^hi^ immature ILCs. Furthermore, this proliferative ILC population emerged early after transplantation of MISTRG mice with human CD34^+^ HSPCs and was also present in human umbilical cord blood. Their “young” ontogeny supports the notion that proliferative CD117^-^CRTH2^-^CD45RA^+^ ILCs may contribute to the ILC compartment in early life. Proliferative CD117^-^CRTH2^-^CD45RA^+^ ILCs were located in both the vascular and tissue compartment of lymphoid and non-lymphoid organs in HSPC-engrafted MISTRG mice but were most abundant in the spleen. Moreover, their spatial distribution is consistent with the idea that proliferative ILCs migrate from the local vasculature into the tissue compartment of the spleen. Overall, these observations suggest a link between ILC proliferation and migration in response to local cues leading to the establishment of tissue-resident ILCs.

We observed that CD117^-^CRTH2^-^CD45RA^+^ ILCs were particularly abundant in the spleen, raising the idea that the spleen constitutes a proliferative niche for ILCs. It is therefore possible that ILC proliferation takes place in the spleen and Ki67^+^ ILCs subsequently enter the circulation to disperse systemically. Alternatively, proliferating ILCs could egress from the bone marrow into the systemic circulation and thereby reach the local spleen vasculature. However, proliferating CD117^-^CRTH2^-^CD45RA^+^ ILCs were almost absent from the bone marrow vasculature, arguing against this possibility. A previous study reported that the spleen environment promotes ILC1 differentiation in humanized mice (45). Furthermore, human CD117^-^CRTH2^-^ ILCs, described as ILC1s, show less transcriptional heterogeneity in lymphoid organs than at mucosal sites (23). ILCs in lymphoid tissues may receive less imprinting by local tissue cues than their counterparts in the mucosa because lymphoid organs are not directly exposed to the outside environment. Therefore, it seems plausible that “ILC1s” in lymphoid organs may be less differentiated. This is consistent with our observation that CD117^-^CRTH2^-^ ILCs in the spleen are highly proliferative and do not have features of mature ILC1s, such as T-BET expression and IFNγ production.

Unexpectedly, we detected a sizeable fraction of Ki67^+^ ILCs within the vasculature in several organs of HSPC-engrafted MISTRG mice as well as in human blood during the neonatal period. In general, circulating (intravascular) immune cells, such as neutrophils, monocytes, and NK cells (36) are thought to represent mobile cells that do not proliferate in steady state. This raises the question whether Ki67^+^ ILCs actively undergo cell division in the circulation because shear forces generated due to rapid blood flow likely make it difficult for cells to divide, at least in large blood vessels. However, the blood flow slows down in smaller vessels and capillaries, resulting in lower shear forces in the local vasculature of organs. Therefore, Ki67^+^ ILCs could divide in the local vasculature when they adhere to the endothelium. Alternatively, intravascular Ki67^+^ ILCs could represent cells that initiated cell division within tissues before entering the systemic circulation. This idea is supported by mouse studies showing that ILC proliferation occurs locally in the intestine before inter-organ trafficking of ILC2s to the lung *via* the circulation (10). Another possibility is that circulating Ki67^+^ ILCs have entered the cell cycle and are primed to complete cell division when they enter tissues from the blood. Consistent with this concept, we found that CD117^-^CRTH2^-^CD45RA^+^ ILCs that expressed intracellular Ki67 protein were mostly in the G1 phase of the cell cycle.

Due to their potent effector functions and strategic positioning, the expansion of ILCs needs to be tightly controlled to avoid tissue pathology and chronic inflammation (56–58). Therefore, understanding the mechanisms of ILC proliferation and migration are of importance in the context of human diseases, where ILCs play a role, such as infection, inflammation, and cancer.

### Limitations of the study

MISTRG mice support human ILC-poiesis and the generation of all ILC subsets, thereby allowing to investigate the biology of human ILCs *in vivo* in a small animal model. One limitation is that human ILCs interact with the mouse tissue environment in MISTRG mice, such as the mouse endothelial cells. However, human ILCs distribute between the vascular and the tissue space within several organs in MISTRG mice, demonstrating that this model supports the migration of human ILCs across the mouse endothelium into tissues. Another limitation is that, besides the spleen, other lymphoid organs, especially lymph nodes, are not fully developed in MISTRG mice, which may affect ILC trafficking and proliferation. Finally, the relatively lymphopenic environment in MISTRG mice could potentially favor increased ILC proliferation. However, this situation is similar to the neonatal period when proliferating ILCs are more abundant in the circulation as shown in our study. Despite the limitations, studying human ILCs *in vivo* in the MISTRG model generates relevant knowledge beyond what is possible to obtain by analyzing ILCs from human blood and tissues *ex vivo*.

## Data availability statement

The datasets presented in this study can be found in online repositories. The names of the repository/repositories and accession number(s) can be found below: https://www.ncbi.nlm.nih.gov/geo/, GSE199965.

## Ethics statement

This study was reviewed and approved by Ethical Review Board at Karolinska Institutet. The patients/participants provided their written informed consent to participate in this study. The animal study was reviewed and approved by Linköping Animal Experimentation Ethics Committee.

## Author contributions

YG and AA designed, performed, and analyzed most experiments. AA contributed to writing the paper. DB analyzed single-cell RNA-sequencing data. NS helped with mouse experiments. JD supervised single-cell RNA-sequencing analysis. TW conceived and supervised the study, acquired funding, designed experiments, analyzed data, and wrote the paper. All authors contributed to the article and approved the submitted version.

## Funding

TW was supported by a faculty-funded career position at Karolinska Institutet (2-1060/2018), a KID grant from Karolinska Institutet (2018-00846), a Junior Investigator Grant from the Center for Innovative Medicine (CIMED) financed by Region Stockholm (2-538/2014), as well as a Project Grant from the Swedish Research Council (2015-02413). JD was supported by grants from the Swedish Research Council (2018-02070) and the Swedish Cancer Society.

## Acknowledgments

We thank Regeneron Pharmaceuticals and Yale University for being able to use MISTRG mice.

## Conflict of interest

The authors declare that the research was conducted in the absence of any commercial or financial relationships that could be construed as a potential conflict of interest.

## Publisher’s note

All claims expressed in this article are solely those of the authors and do not necessarily represent those of their affiliated organizations, or those of the publisher, the editors and the reviewers. Any product that may be evaluated in this article, or claim that may be made by its manufacturer, is not guaranteed or endorsed by the publisher.

## Supplementary material

Supplementary Table 1 List of antibodies used for flow cytometry.

Supplementary Table 2 Genes in each human ILC cluster from the spleens of HSPC-engrafted MISTRG mice as defined by single-cell RNA-sequencing.

## Abbreviations

HSPCs, human hematopoietic stem and progenitor cells; IFNγ, interferon gamma; IL, interleukin; ILCs, innate lymphoid cells; ILCPs, innate lymphoid cell precursors; IV, intravenous; Lin, lineage; NK, natural killer; TCF-1, T cell factor-1; UMAP, Uniform Manifold Approximation and Projection.

## References

[1] Artis D Spits H. The biology of innate lymphoid cells. Nature (2015) 517(7534):293–301. doi: 10.1038/nature14189 25592534

[2] Eberl G Colonna M Di Santo JP McKenzie AN. Innate lymphoid cells. innate lymphoid cells: a new paradigm in immunology. Science (2015) 348(6237):aaa6566. doi: 10.1126/science.aaa6566 25999512 PMC5658207

[3] Vivier E Artis D Colonna M Diefenbach A Di Santo JP Eberl G. Innate lymphoid cells: 10 years on. Cell (2018) 174(5):1054–66. doi: 10.1016/j.cell.2018.07.017 30142344

[4] Colonna M. Innate lymphoid cells: Diversity, plasticity, and unique functions in immunity. Immunity (2018) 48(6):1104–17. doi: 10.1016/j.immuni.2018.05.013 PMC6344351 29924976

[5] Bal SM Golebski K Spits H. Plasticity of innate lymphoid cell subsets. Nat Rev Immunol (2020) 20(9):552–65. doi: 10.1038/s41577-020-0282-9 32107466

[6] Kotas ME Locksley RM. Why innate lymphoid cells? Immunity (2018) 48(6):1081–90. doi: 10.1016/j.immuni.2018.06.002 PMC6145487 29924974

[7] Hazenberg MD Spits H. Human innate lymphoid cells. Blood (2014) 124(5):700–9. doi: 10.1182/blood-2013-11-427781 24778151

[8] Gasteiger G Fan X Dikiy S Lee SY Rudensky AY. Tissue residency of innate lymphoid cells in lymphoid and nonlymphoid organs. Science (2015) 350(6263):981–5. doi: 10.1126/science.aac9593 PMC4720139 26472762

[9] Emgard J Kammoun H Garcia-Cassani B Chesne J Parigi SM Jacob JM. Oxysterol sensing through the receptor GPR183 promotes the lymphoid-Tissue-Inducing function of innate lymphoid cells and colonic inflammation. Immunity (2018) 48(1):120–32.e8. doi: 10.1016/j.immuni.2017.11.020 29343433 PMC5772175

[10] Huang Y Mao K Chen X Sun MA Kawabe T Li W. S1P-dependent interorgan trafficking of group 2 innate lymphoid cells supports host defense. Science (2018) 359(6371):114–9. doi: 10.1126/science.aam5809 PMC6956613 29302015

[11] Zeis P Lian M Fan X Herman JS Hernandez DC Gentek R. *In situ* maturation and tissue adaptation of type 2 innate lymphoid cell progenitors. Immunity (2020) 53(4):775–92.e9. doi: 10.1016/j.immuni.2020.09.002 33002412 PMC7611573

[12] Pearson C Thornton EE McKenzie B Schaupp AL Huskens N Griseri T. ILC3 GM-CSF production and mobilisation orchestrate acute intestinal inflammation. Elife (2016) 5:e10066. doi: 10.7554/eLife.10066 26780670 PMC4733039

[13] Puttur F Denney L Gregory LG Vuononvirta J Oliver R Entwistle LJ. Pulmonary environmental cues drive group 2 innate lymphoid cell dynamics in mice and humans. Sci Immunol (2019) 4(36):eaav7638. doi: 10.1126/sciimmunol.aav7638 31175176 PMC6744282

[14] Kim MH Taparowsky EJ Kim CH. Retinoic acid differentially regulates the migration of innate lymphoid cell subsets to the gut. Immunity (2015) 43(1):107–19. doi: 10.1016/j.immuni.2015.06.009 PMC4511719 26141583

[15] Kastele V Mayer J Lee ES Papazian N Cole JJ Cerovic V. Intestinal-derived ILCs migrating in lymph increase IFNgamma production in response to salmonella typhimurium infection. Mucosal Immunol (2021) 14(3):717–27. doi: 10.1038/s41385-020-00366-3 PMC8075955 33414524

[16] Dutton EE Gajdasik DW Willis C Fiancette R Bishop EL Camelo A. Peripheral lymph nodes contain migratory and resident innate lymphoid cell populations. Sci Immunol (2019) 4(35):eaau8082. doi: 10.1126/sciimmunol.aau8082 31152090 PMC7018521

[17] Ricardo-Gonzalez RR Schneider C Liao C Lee J Liang HE Locksley RM. Tissue-specific pathways extrude activated ILC2s to disseminate type 2 immunity. J Exp Med (2020) 217(4):e20191172. doi: 10.1084/jem.20191172 32031571 PMC7144525

[18] Cautivo KM Matatia PR Lizama CO Mroz NM Dahlgren MW Yu X. Interferon gamma constrains type 2 lymphocyte niche boundaries during mixed inflammation. Immunity (2022) 55(2):254–71.e7. doi: 10.1016/j.immuni.2021.12.014 35139352 PMC8852844

[19] Ricardo-Gonzalez RR Van Dyken SJ Schneider C Lee J Nussbaum JC Liang HE. Tissue signals imprint ILC2 identity with anticipatory function. Nat Immunol (2018) 19(10):1093–9. doi: 10.1038/s41590-018-0201-4 PMC6202223 30201992

[20] Kim CH Hashimoto-Hill S Kim M. Migration and tissue tropism of innate lymphoid cells. Trends Immunol (2016) 37(1):68–79. doi: 10.1016/j.it.2015.11.003 26708278 PMC4744800

[21] Willinger T. Metabolic control of innate lymphoid cell migration. Front Immunol (2019) 10:2010. doi: 10.3389/fimmu.2019.02010 31507605 PMC6713999

[22] Simoni Y Fehlings M Kloverpris HN McGovern N Koo SL Loh CY. Human innate lymphoid cell subsets possess tissue-type based heterogeneity in phenotype and frequency. Immunity (2017) 46(1):148–61. doi: 10.1016/j.immuni.2016.11.005 PMC7612935 27986455

[23] Yudanin NA Schmitz F Flamar AL Thome JJC Tait Wojno E Moeller JB. Spatial and temporal mapping of human innate lymphoid cells reveals elements of tissue specificity. Immunity (2019) 50(2):505–19.e4. doi: 10.1016/j.immuni.2019.01.012 30770247 PMC6594374

[24] Lim AI Li Y Lopez-Lastra S Stadhouders R Paul F Casrouge A. Systemic human ILC precursors provide a substrate for tissue ILC differentiation. Cell (2017) 168(6):1086–100.e10. doi: 10.1016/j.cell.2017.02.021 28283063

[25] Bar-Ephraim YE Koning JJ Burniol Ruiz E Konijn T Mourits VP Lakeman KA. CD62L is a functional and phenotypic marker for circulating innate lymphoid cell precursors. J Immunol (2019) 202(1):171–82. doi: 10.4049/jimmunol.1701153 30504420

[26] Nagasawa M Heesters BA Kradolfer CMA Krabbendam L Martinez-Gonzalez I de Bruijn MJW. KLRG1 and NKp46 discriminate subpopulations of human CD117(+)CRTH2(-) ILCs biased toward ILC2 or ILC3. J Exp Med (2019) 216(8):1762–76. doi: 10.1084/jem.20190490 PMC6683990 31201208

[27] Rongvaux A Willinger T Martinek J Strowig T Gearty SV Teichmann LL. Development and function of human innate immune cells in a humanized mouse model. Nat Biotechnol (2014) 32(4):364–72. doi: 10.1038/nbt.2858 PMC4017589 24633240

[28] Willinger T Rongvaux A Strowig T Manz MG Flavell RA. Improving human hemato-lymphoid-system mice by cytokine knock-in gene replacement. Trends Immunol (2011) 32(7):321–7. doi: 10.1016/j.it.2011.04.005 21697012

[29] Rongvaux A Takizawa H Strowig T Willinger T Eynon EE Flavell RA. Human hemato-lymphoid system mice: current use and future potential for medicine. Annu Rev Immunol (2013) 31:635–74. doi: 10.1146/annurev-immunol-032712-095921 PMC4120191 23330956

[30] Alisjahbana A Mohammad I Gao Y Evren E Ringqvist E Willinger T. Human macrophages and innate lymphoid cells: Tissue-resident innate immunity in humanized mice. Biochem Pharmacol (2020) 174:113672. doi: 10.1016/j.bcp.2019.113672 31634458

[31] Alisjahbana A Gao Y Sleiers N Evren E Brownlie D von Kries A. CD5 surface expression marks intravascular human innate lymphoid cells that have a distinct ontogeny and migrate to the lung. Front Immunol (2021) 12:752104. doi: 10.3389/fimmu.2021.752104 34867984 PMC8640955

[32] Deng K Pertea M Rongvaux A Wang L Durand CM Ghiaur G. Broad CTL response is required to clear latent HIV-1 due to dominance of escape mutations. Nature (2015) 517(7534):381–5. doi: 10.1038/nature14053 PMC4406054 25561180

[33] Evren E Ringqvist E Tripathi KP Sleiers N Rives IC Alisjahbana A. Distinct developmental pathways from blood monocytes generate human lung macrophage diversity. Immunity (2021) 54(2):259–75.e7. doi: 10.1016/j.immuni.2020.12.003 33382972

[34] Bjorklund AK Forkel M Picelli S Konya V Theorell J Friberg D. The heterogeneity of human CD127(+) innate lymphoid cells revealed by single-cell RNA sequencing. Nat Immunol (2016) 17(4):451–60. doi: 10.1038/ni.3368 26878113

[35] van der Ploeg EK Golebski K van Nimwegen M Fergusson JR Heesters BA Martinez-Gonzalez I. Steroid-resistant human inflammatory ILC2s are marked by CD45RO and elevated in type 2 respiratory diseases. Sci Immunol (2021) 6(55):eabd3489. doi: 10.1126/sciimmunol.abd3489 33514640

[36] Collins PL Cella M Porter SI Li S Gurewitz GL Hong HS. Gene regulatory programs conferring phenotypic identities to human NK cells. Cell (2019) 176(1-2):348–60.e12. doi: 10.1016/j.cell.2018.11.045 30595449 PMC6329660

[37] Friedrich C Taggenbrock R Doucet-Ladeveze R Golda G Moenius R Arampatzi P. Effector differentiation downstream of lineage commitment in ILC1s is driven by hobit across tissues. Nat Immunol (2021) 22(10):1256–67. doi: 10.1038/s41590-021-01013-0 PMC7611762 34462601

[38] Cherrier DE Serafini N Di Santo JP. Innate lymphoid cell development: A T cell perspective. Immunity (2018) 48(6):1091–103. doi: 10.1016/j.immuni.2018.05.010 29924975

[39] Constantinides MG McDonald BD Verhoef PA Bendelac A. A committed precursor to innate lymphoid cells. Nature (2014) 508(7496):397–401. doi: 10.1038/nature13047 24509713 PMC4003507

[40] Juelke K Romagnani C. Differentiation of human innate lymphoid cells (ILCs). Curr Opin Immunol (2016) 38:75–85. doi: 10.1016/j.coi.2015.11.005 26707651

[41] Scoville SD Freud AG Caligiuri MA. Cellular pathways in the development of human and murine innate lymphoid cells. Curr Opin Immunol (2019) 56:100–6. doi: 10.1016/j.coi.2018.11.003 PMC7285385 30579240

[42] Vely F Barlogis V Vallentin B Neven B Piperoglou C Ebbo M. Evidence of innate lymphoid cell redundancy in humans. Nat Immunol (2016) 17(11):1291–9. doi: 10.1038/ni.3553 PMC5074366 27618553

[43] Bar-Ephraim YE Cornelissen F Papazian N Konijn T Hoogenboezem RM Sanders MA. Cross-tissue transcriptomic analysis of human secondary lymphoid organ-residing ILC3s reveals a quiescent state in the absence of inflammation. Cell Rep (2017) 21(3):823–33. doi: 10.1016/j.celrep.2017.09.070 29045847

[44] Hernandez DC Juelke K Muller NC Durek P Ugursu B Mashreghi MF. An in vitro platform supports generation of human innate lymphoid cells from CD34(+) hematopoietic progenitors that recapitulate ex vivo identity. Immunity (2021) 54(10):2417–32.e5. doi: 10.1016/j.immuni.2021.07.019 34453879

[45] Cella M Gamini R Secca C Collins PL Zhao S Peng V. Subsets of ILC3-ILC1-like cells generate a diversity spectrum of innate lymphoid cells in human mucosal tissues. Nat Immunol (2019) 20(8):980–91. doi: 10.1038/s41590-019-0425-y PMC6685551 31209406

[46] Xu W Cherrier DE Chea S Vosshenrich C Serafini N Petit M. An Id2(RFP)-reporter mouse redefines innate lymphoid cell precursor potentials. Immunity (2019) 50(4):1054–68.e3. doi: 10.1016/j.immuni.2019.02.022 30926235 PMC6477155

[47] Walker JA Clark PA Crisp A Barlow JL Szeto A Ferreira ACF. Polychromic reporter mice reveal unappreciated innate lymphoid cell progenitor heterogeneity and elusive ILC3 progenitors in bone marrow. Immunity (2019) 51(1):104–18.e7. doi: 10.1016/j.immuni.2019.05.002 31128961 PMC6642165

[48] Pais Ferreira D Silva JG Wyss T Fuertes Marraco SA Scarpellino L Charmoy M. Central memory CD8(+) T cells derive from stem-like Tcf7(hi) effector cells in the absence of cytotoxic differentiation. Immunity (2020) 53(5):985–1000.e11. doi: 10.1016/j.immuni.2020.09.005 33128876

[49] Ji Y Pos Z Rao M Klebanoff CA Yu Z Sukumar M. Repression of the DNA-binding inhibitor Id3 by blimp-1 limits the formation of memory CD8+ T cells. Nat Immunol (2011) 12(12):1230–7. doi: 10.1038/ni.2153 PMC3226770 22057288

[50] Shan Q Hu SS Zhu S Chen X Badovinac VP Peng W. Tcf1 preprograms the mobilization of glycolysis in central memory CD8(+) T cells during recall responses. Nat Immunol (2022) 23(3):386–98. doi: 10.1038/s41590-022-01131-3 PMC8904300 35190717

[51] Bennstein SB Scherenschlich N Weinhold S Manser AR Noll A Raba K. Transcriptional and functional characterization of neonatal circulating innate lymphoid cells. Stem Cells Transl Med (2021) 10(6):867–82. doi: 10.1002/sctm.20-0300 PMC8133339 33475258

[52] Bennstein SB Weinhold S Manser AR Scherenschlich N Noll A Raba K. Umbilical cord blood-derived ILC1-like cells constitute a novel precursor for mature KIR(+)NKG2A(-) NK cells. Elife (2020) 9:e55232. doi: 10.7554/eLife.55232 32657756 PMC7358013

[53] Mazzurana L Czarnewski P Jonsson V Wigge L Ringner M Williams TC. Tissue-specific transcriptional imprinting and heterogeneity in human innate lymphoid cells revealed by full-length single-cell RNA-sequencing. Cell Res (2021) 31(5):554–68. doi: 10.1038/s41422-020-00445-x PMC8089104 33420427

[54] Escobar G Mangani D Anderson AC. T Cell factor 1: A master regulator of the T cell response in disease. Sci Immunol (2020) 5(53):eabb9726. doi: 10.1126/sciimmunol.abb9726 33158974 PMC8221367

[55] Zhao X Shan Q Xue HH. TCF1 in T cell immunity: a broadened frontier. Nat Rev Immunol (2021) 22(3):147–57. doi: 10.1038/s41577-021-00563-6 34127847

[56] Sonnenberg GF Artis D. Innate lymphoid cells in the initiation, regulation and resolution of inflammation. Nat Med (2015) 21(7):698–708. doi: 10.1038/nm.3892 26121198 PMC4869856

[57] Branzk N Gronke K Diefenbach A. Innate lymphoid cells, mediators of tissue homeostasis, adaptation and disease tolerance. Immunol Rev (2018) 286(1):86–101. doi: 10.1111/imr.12718 30294961

[58] Ebbo M Crinier A Vely F Vivier E. Innate lymphoid cells: major players in inflammatory diseases. Nat Rev Immunol (2017) 17(11):665–78. doi: 10.1038/nri.2017.86 28804130

