# Supplementary material for: A single-cell map of vascular and tissue lymphocytes identifies proliferative TCF-1+ human innate lymphoid cells
Source: Front Immunol. 2022 Jul 27;13:902881. doi: 10.3389/fimmu.2022.902881 (PMC9364238; doi:10.3389/fimmu.2022.902881)
Supplement: Supplementary file 3 [file DataSheet_1.pdf]

Figure S1

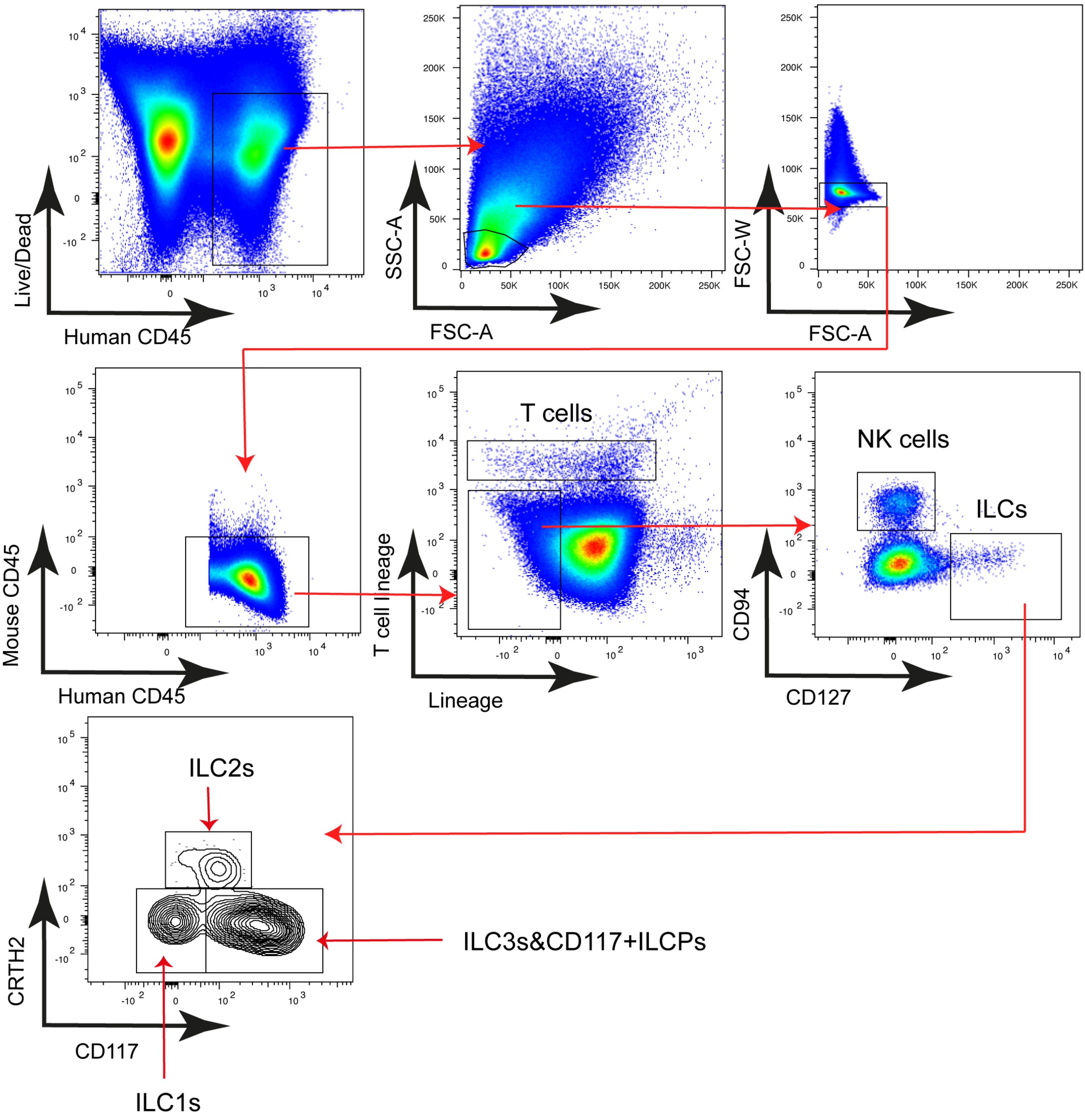

SUPPLEMENTARY FIGURE 1 Gating strategy for the flow cytometric analysis of human ILCs in HSPC-engrafted MISTRG mice . Total ILCs were gated as viable human $CD45^{+}CD127^{+}CD94^{-}CD3^{-}TCR\alpha\beta^{-}$ cells that were negative for Lineage markers (CD11c, CD14, CD19, CD123, FceRI) and mouse CD45. Data are representative of two independent experiments using MISTRG mice engrafted with different pools of HSPCs.

Figure S2

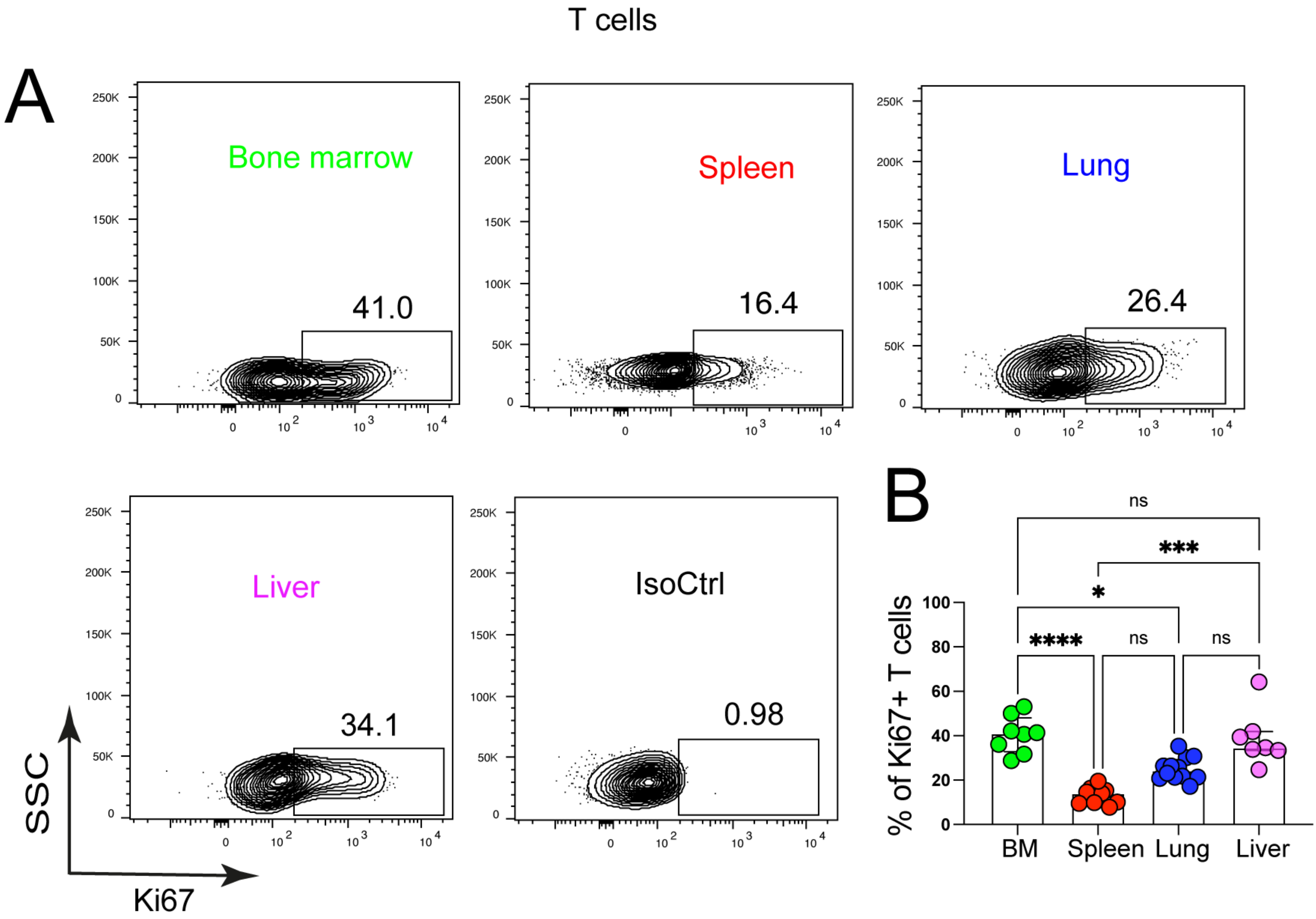

SUPPLEMENTARY FIGURE 2 Proliferative status of human T lymphocytes in HSPC-engrafted MISTRG mice. **(A and B)** Intracellular Ki67 expression by human T cells from HSPC-engrafted MISTRG mice as determined by flow cytometry. Isotype control (IsoCtrl) staining is shown for the lung. T cells were gated as human $CD45^{+}CD3^{+}TCR\alpha\beta^{+}$ cells as in Supplementary Figure 1. Frequencies of $Ki67^{+}$ T cells in the indicated organs of HSPC-engrafted MISTRG mice (n = 7-12) are shown in (B). n.s., not significant; **, *P* <0.01; ***, P <0.001; ****, *P* <0.0001 by one-way ANOVA, Tukey's post -test. Data are represented as mean ± SEM. Data are from two independent experiments using MISTRG mice engrafted with different pools of HSPCs.

Figure S3

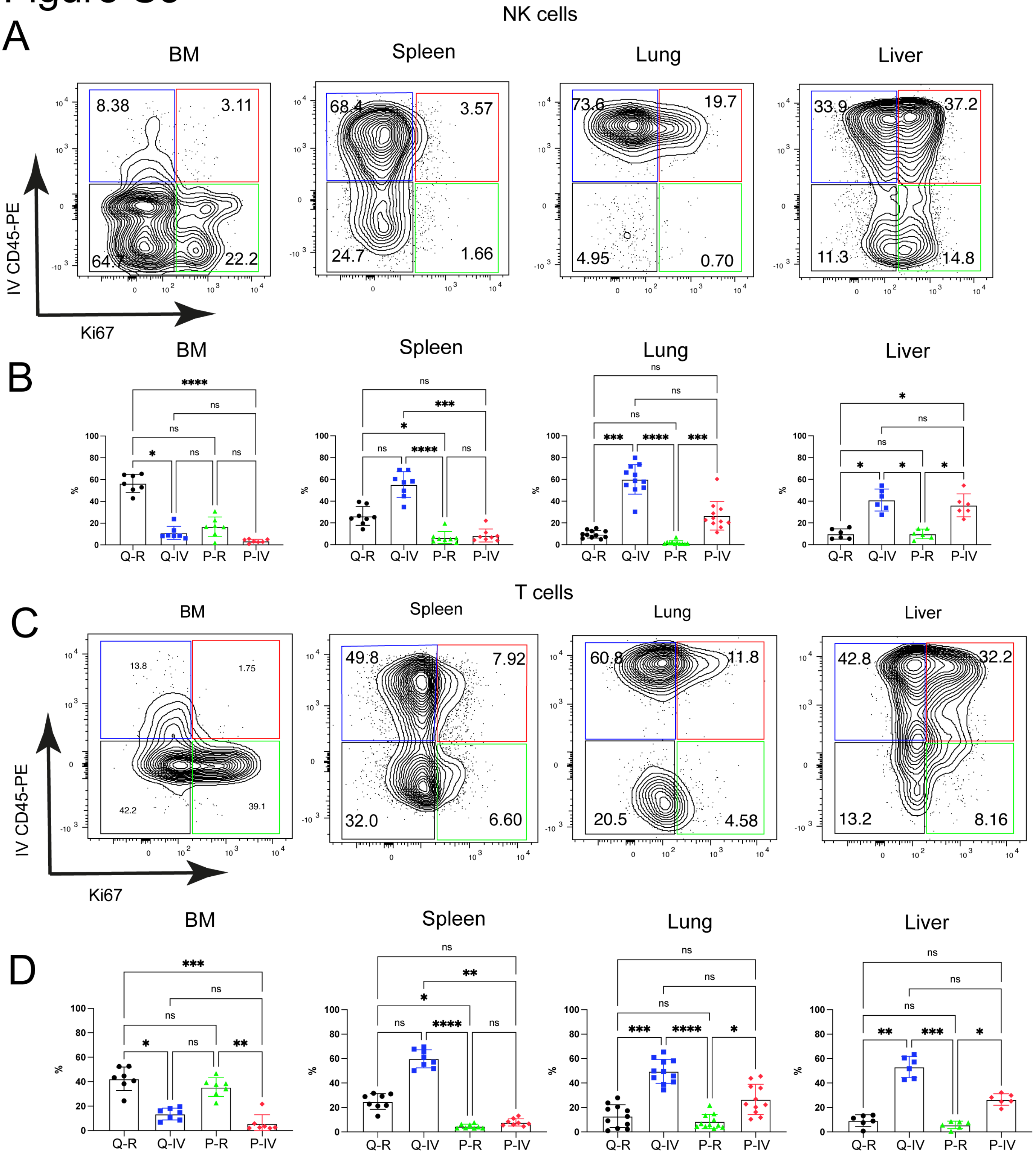

SUPPLEMENTARY FIGURE 3 Intravascular and extravascular distribution of proliferating T cells within organs in HSPC -engrafted MISTRG mice. **(A)** Flow cytometry analysis of intravascular versus extravascular and quiescent versus proliferative human NK cells in the indicated organs of HSPC -engrafted MISTRG mice as in Figure 2 . NK cells were gated as human CD45$^+$CD127$^-$CD94$^+$CD3$^-$TCRαβ$^-$Lin$^-$ cells as in Supplementary Figure 1. **(B)** Frequencies of quiescent tissue -resident (Q -R), quiescent intravascular (Q -IV), proliferative tissue-resident (P-R), and proliferative intravascular (P-IV) NK cells in the indicated organs (n = 6-11) as determined in (A). **(C)** Flow cytometry analysis of intravascular versus extravascular and quiescent versus proliferative human T cells in the indicated organs of HSPC -engrafted MISTRG mice as in Figure 2. T cells were gated as human CD45$^+$CD3$^+$TCRαβ$^+$ cells as in Supplementary Figure 1. **(D)** Frequencies of quiescent tissue -resident (Q -R), quiescent intravascular (Q-IV), proliferative tissue-resident (P-R), and proliferative intravascular (P-IV) NK cells in the indicated organs (n = 6-11) as determined in (C). n.s., not significant; **, *P* <0.01; ***, P <0.001; ****,*P* <0.0001 by one-way ANOVA, Tukey's post -test. Data represent mean ± SEM and are representative of two independent experiments using MISTRG mice engrafted with different pools of HSPCs.

Figure S4

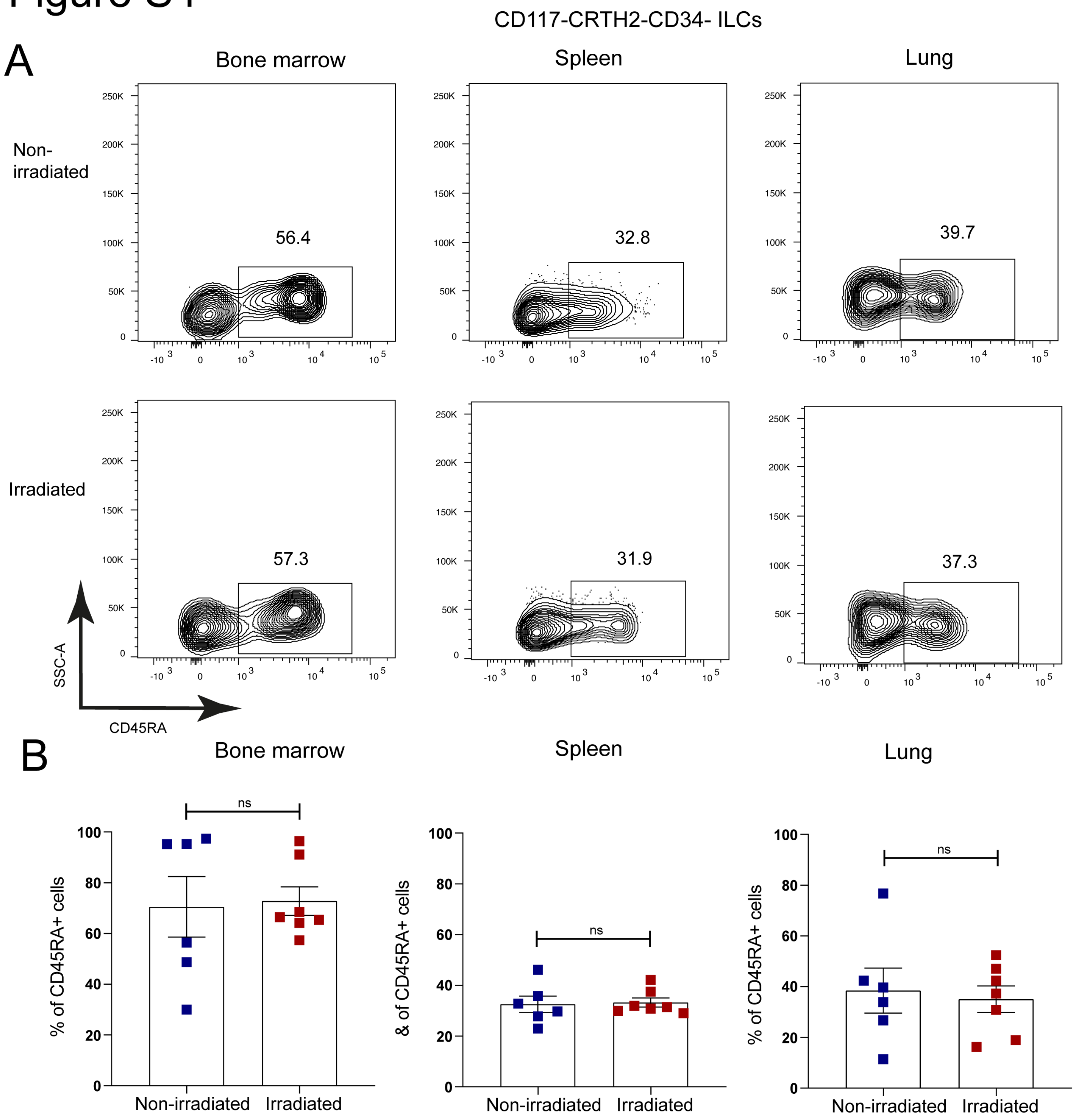

SUPPLEMENTARY FIGURE 4 CD117$^-$CRTH2$^-$CD45RA$^+$ ILCs inhabit a radio-insensitive niche in MISTRG mice. **(A)** Flow cytometry of CD117$^-$CRTH2$^-$CD34$^-$ ILCs in bone marrow, spleen, and lung of HSPC-engrafted MISTRG mice that were either not irradiated or irradiated before transplantation with human CD34$^+$ HSPCs. ILCs were gated as human CD45$^+$CD127$^+$CD94$^-$CD3$^-$TCRαβ$^-$Lin$^-$ cells as in Supplementary Figure 1. **(B)** Frequencies of CD117$^-$CRTH2$^-$CD34$^-$CD45RA$^+$ ILCs in bone marrow, spleen, and lung of HSPC-engrafted MISTRG mice that were either not irradiated or irradiated before transplantation with human CD34$^+$ HSPCs (n = 6-7). n.s., not significant by Students *t* test. Data represent mean ± SEM and are representative of two independent experiments using MISTRG mice engrafted with different pools of HSPCs.

Figure S5

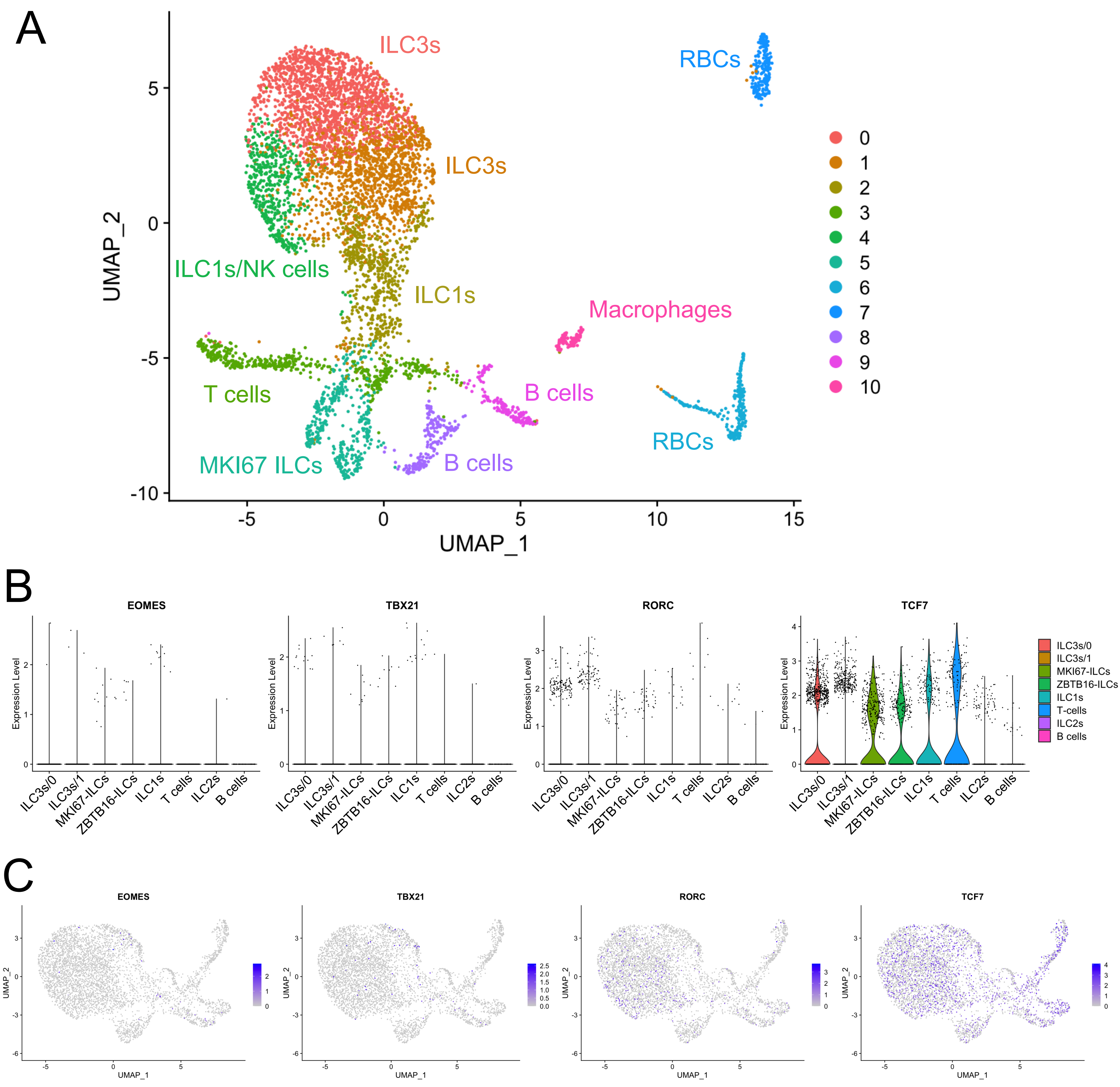

SUPPLEMENTARY FIGURE 5 Single-cell RNA-sequencing of human ILCs from spleens of HSPC-engrafted MISTRG mice. (**A**) The UMAP shows 5,853 cells, consisting of human ILC clusters as well as contaminating clusters of B cells, macrophages, and red blood cells (RBCs). (**B and C**) Violin plots (B) and Feature plots (C) showing expression of transcription factors in the human ILC clusters from the UMAP in Figure 7B. Data are from one single-cell RNA-sequencing experiment with spleen cells pooled from ten MISTRG mice engrafted with three pooled batches of human $CD34^{+}$ cells.

Figure S6

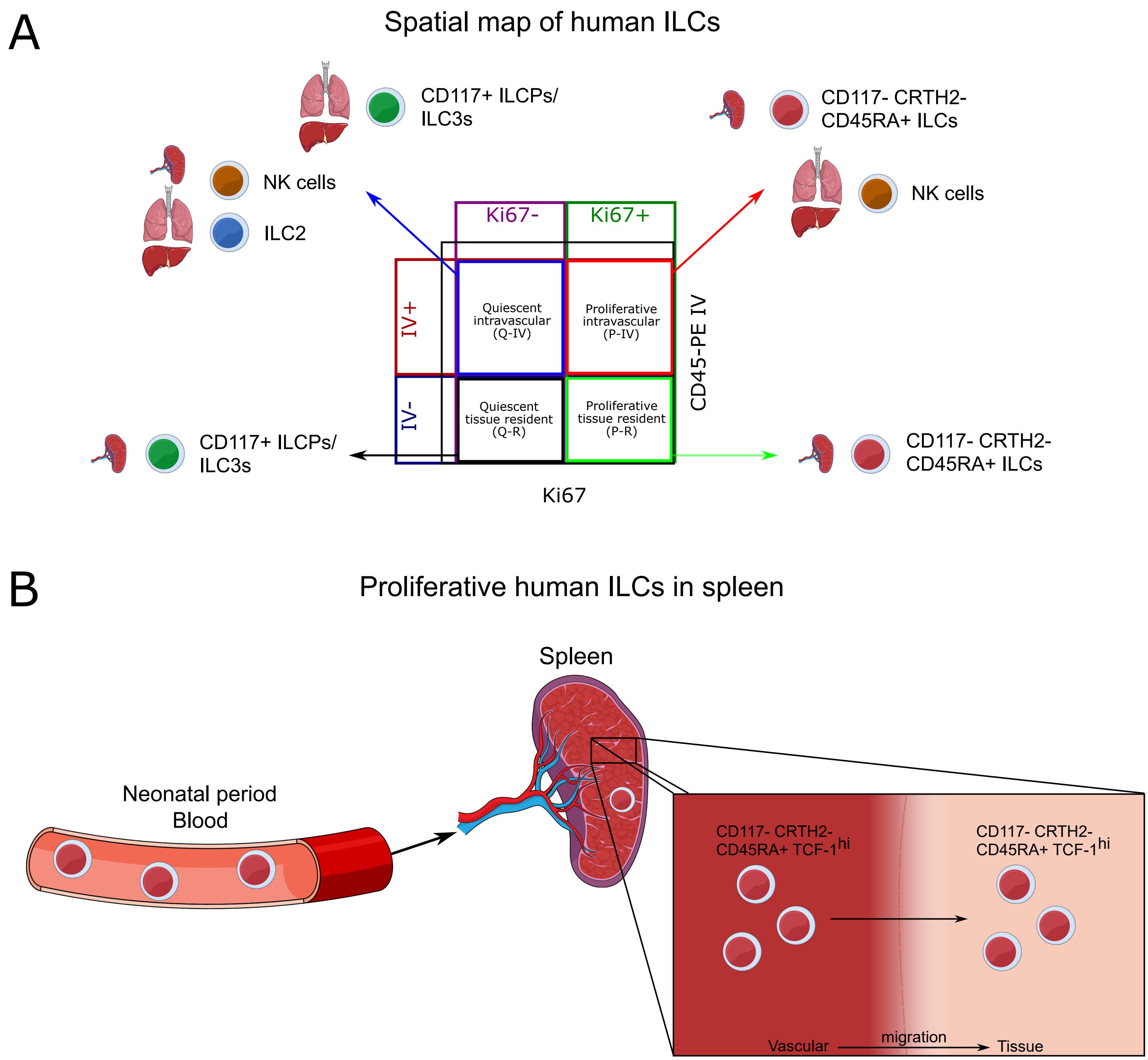

SUPPLEMENTARY FIGURE 6 Graphical Abstract . (**A**) Spatial map of human ILCs in HSPC-engrafted MISTRG mice. Four different categories of ILCs are distinguished by proliferative status and anatomical localization within lymphoid and non-lymphoid organs. The main ILC subsets within each category and organ are indicated. (**B**) Model of proliferative human ILCs in the spleen of HSPC -engrafted MISTRG mice. During the neonatal period, circulating CD117$^-$CRTH2$^-$CD45RA$^+$ proliferative ILCs occupy the intravascular compartment of the spleen. These proliferative ILCs express the transcription factor TCF-1 and migrate into the tissue compartment of the spleen. Parts of the figure were adapted from Mind the Graph.
